# *CARD11* gain-of-function mutation drives cell-autonomous accumulation of PD-1^+^ ICOS^high^ activated T cells, T-follicular, T-regulatory and T-follicular regulatory cells

**DOI:** 10.3389/fimmu.2023.1095257

**Published:** 2023-03-07

**Authors:** Etienne Masle-Farquhar, Yogesh Jeelall, Jacqueline White, Julia Bier, Elissa K. Deenick, Robert Brink, Keisuke Horikawa, Christopher Carl Goodnow

**Affiliations:** ^1^ Garvan Institute of Medical Research, Sydney, NSW, Australia; ^2^ School of Clinical Medicine, St Vincent’s Healthcare Clinical, Faculty of Medicine and Health, University of New South Wales, Sydney, NSW, Australia; ^3^ John Curtin School of Medical Research, Immunology Department, The Australian National University, Canberra, ACT, Australia; ^4^ Cellular Genomics Futures Institute, University of New South Wales, Sydney, Australia

**Keywords:** CARD11, mutation, gain-of-function, regulatory T cell, follicular T cell, germinal center, lymphoproliferation, lymphoma

## Abstract

**Introduction:**

Germline CARD11 gain-of-function (GOF) mutations cause B cell Expansion with NF-κB and T cell Anergy (BENTA) disease, whilst somatic GOF CARD11 mutations recur in diffuse large B cell lymphoma (DLBCL) and in up to 30% of the peripheral T cell lymphomas (PTCL) adult T cell leukemia/lymphoma (ATL), cutaneous T cell lymphoma (CTCL) and Sezary Syndrome. Despite their frequent acquisition by PTCL, the T cell-intrinsic effects of CARD11 GOF mutations are poorly understood.

**Methods:**

Here, we studied B and T lymphocytes in mice with a germline Nethyl-N-nitrosourea (ENU)-induced Card11^M365K^ mutation identical to a mutation identified in DLBCL and modifying a conserved region of the CARD11 coiled-coil domain recurrently mutated in DLBCL and PTCL.

**Results and discussion:**

Our results demonstrate that CARD11.M365K is a GOF protein that increases B and T lymphocyte activation and proliferation following antigen receptor stimulation. Germline Card11^M365K^ mutation was insufficient alone to cause B or T-lymphoma, but increased accumulation of germinal center (GC) B cells in unimmunized and immunized mice. Card11^M365K^ mutation caused cell-intrinsic over-accumulation of activated T cells, T regulatory (T_REG_), T follicular (T_FH_) and T follicular regulatory (T_FR_) cells expressing increased levels of ICOS, CTLA-4 and PD-1 checkpoint molecules. Our results reveal CARD11 as an important, cell-autonomous positive regulator of T_FH_, T_REG_ and T_FR_ cells. They highlight T cell-intrinsic effects of a GOF mutation in the CARD11 gene, which is recurrently mutated in T cell malignancies that are often aggressive and associated with variable clinical outcomes.

## 1 Introduction

Nuclear factor-κB (NF-κB) signaling downstream of the B or T cell receptor (BCR or TCR) requires the MAGUK family member Caspase Recruitment Domain-containing protein 11 (CARD11) (1) and its phosphorylation by protein kinase C (PKC) (2, 3). CARD11 forms a scaffold to recruit signaling partners B cell lymphoma/leukemia 10 (BCL10) and mucosa-associated lymphoid tissue lymphoma translocation protein 1 (MALT1) (4) into the CBM complex, which relays antigen receptor signals to NF-κB and activator protein 1 (AP1)-cJUN (1, 5–8).

CARD11 function is required for B and T cell immunity. In mice, germline CARD11 deficiency or loss-of-function (LOF) disrupt B cell development and humoral immunity (9–12), disrupt T cell NF-κB activation, proliferation and IL-2 production following TCR and CD28 stimulation (9–12) and perturb thymic (though not peripheral [13)] Treg development in response to TCR (13, 14) and IL-2 signaling (15). In hypomorphic *Card11* “unmodulated” mice, partial reduction of TCR-NF-κB signaling causes a recessive phenotype of hyper-IgE and atopy (10) driven by reduced Treg numbers and a gradual and selective expansion of IL-4-producing T_H_2 cells (16). Similarly, dominant-negative heterozygous *CARD11* mutations in humans skew T cells towards a T_H_2 phenotype and cause severe atopic disease (17, 18) as well as common variable immunodeficiency (CVID), cutaneous viral infections, lung disease and characteristics reminiscent of immune dysregulation, polyendocrinopathy, enteropathy, X-linked (IPEX) (19). Homozygous germline *CARD11* truncations cause combined immunodeficiency, a developmental block at the B cell transitional stage and hypogammaglobulinemia (20, 21).

CARD11 gain-of-function (GOF) mutations also cause pathology. Heterozygous germline GOF *CARD11* mutations cause B cell Expansion with NF-κB and T cell Anergy (BENTA), a rare monogenic disease characterized by B cell lymphocytosis and aspects of primary immunodeficiency including recurrent and opportunistic infections (22–29). In addition, *somatic CARD11* GOF mutations recur in germinal center B cell-type (GCB) and activated B cell-like (ABC)-diffuse large B cell lymphoma (DLBCL) (30, 31), an aggressive subset of DLBCL (32) characterized by constitutive NF-κB activation (33, 34). ABC-DLBCL harbor recurrent somatic GOF mutations in the BCR-NF-κB pathway (30, 35), including *CARD11* (31), and require CARD11 and the CBM complex for their survival *in vitro* (34). The recurrent B-lymphoma *CARD11* mutations cluster in the CARD and coiled-coil (CC) domains and disrupt the intrinsic ‘auto-inhibited’ conformation of CARD11 (36), uncoupling it from activating signals and causing it to form aggregates with other CARD11 proteins, MALT1 and BCL10, and thus activate NF-κB and AP1/cJUN (31, 36, 37).

*CARD11* is the fourth most mutated gene in adult T cell leukemia/lymphoma (ATL (38, 39);), a CD4 T cell neoplasm (40) that arises exclusively in individuals previously infected with Human T cell Lymphotropic Virus type 1 (HTLV-1 (41, 42);). Up to 90% of ATL (38) harbor somatic mutations in TCR-NFκB signaling molecules including *PLCG1, PRKCB, VAV1* and *CARD11*. 24% of ATL harbor *CARD11* mutations predicted to constitutively activate CARD11, and clustered in the CC domain or around the E626 hotspot in the PKC-responsive inhibitory domain (38). Moreover, 12% of ATL harbor *CARD11* gene amplifications and 8% harbor small intragenic deletions in the CARD11 inhibitory domain (38). Up to 22.5% of Cutaneous T Cell Lymphomas (CTCL) harbor *CARD11* amplifications (43) and up to 24% of Sezary syndrome, the aggressive form of CTCL, harbor *CARD11* GOF mutations clustered in the CC domain or surrounding the E626 hotspot (44–46). PTCL are often associated with very poor outcomes (47, 48) and are thought derived from activated CD4 and T_REG_ cells (49, 50).

The study of *CARD11* mutations in B- and T-lymphomas is complicated by the many genomic alterations acquired by these cancer cells (38, 51). Previous studies have used mouse models to reveal B cell-intrinsic effects of GOF CARD11.L232LI (52), CARD11.L251P (53), CARD11.K215M or CARD11.E134G (54). These studies reported variable effects on B cells of different *Card11* mutations, and the wide spectrum of GOF *CARD11* mutations have diverse biochemical effects (55). The graded, variable effects of hypomorphic *Card11* mutation within distinct T cell populations could not be predicted from knockout studies (16). The effects of *hypermorphic CARD11* mutations are thus similarly hard to predict *a priori*. Collectively, the above observations highlight open questions regarding qualitative differences in NF-κB activation by *CARD11* mutations, and possible discontinuity in the graded effects of *CARD11* GOF mutations within different cell types. Crucially, to our knowledge, no studies have reported T cell-intrinsic effects of CARD11 GOF, despite the striking recurrence of somatic *CARD11* GOF mutations in PTCL.

**Figure 1:**
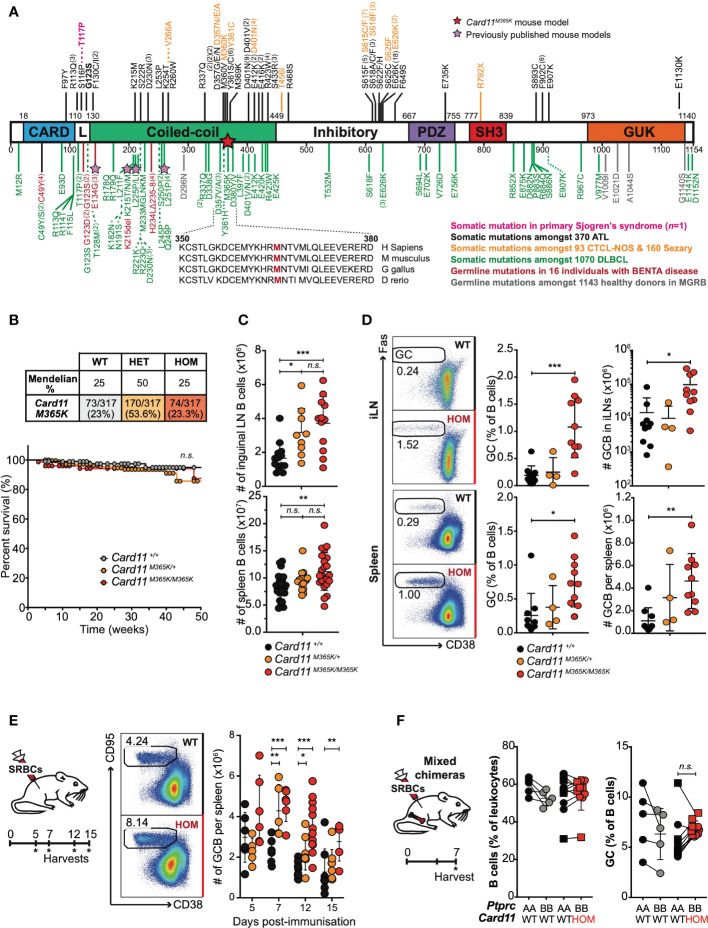
Gain-of-function *Card11^M365K^* germline mutant mice have increased numbers of germinal center B cells. **(A)**. Schematic of CARD11 protein domains showing location of germline GOF mutations in healthy donors from the MGRB database (grey) or individuals with BENTA disease (red (23–29);), and somatic GOF mutations identified in adult T cell leukemia/lymphoma (ATL (38, 56–58); black), cutaneous T cell lymphoma not otherwise-specified (CTCL-NOS) or Sezary syndrome (orange (43–46, 59–61);), diffuse large B cell lymphoma (DLBCL (30, 31, 51, 62–64); green) or in one individual with primary Sjögren’s syndrome (pink (65);). Stars indicate the *Card11^M365K^* or other, previously published (52–54), mouse models of CARD11 GOF. **(B)**. Top, Expected and observed numbers and percentages of offspring of the indicated genotypes from intercrossed heterozygous parents. Statistical testing for no difference relative to an expected 1WT:2HET:1HOM ratio *p*=0.4328 by Chi-Square test with *n*=2 degrees of freedom. Bottom, Kaplan-Meier survival curve for mice of the indicated genotypes, calculated using the product-limit method accounting for censored mice. **(C)**. Symbols denote total number of B cells in the inguinal lymph nodes (top) or spleen (bottom) from individual mice of the indicated genotypes. **(D)**. Left, Representative flow cytometric analysis showing the percentage of CD38^low^ CD95^+^ germinal center (GC) B cells gated on splenic B cells in non-immunized mice. Right, percentage or total number of GC B cells in the inguinal lymph nodes (iLN; top) or spleen (bottom) from mice of the indicated genotypes. **(E)**. Left: Schematic of experimental workflow. Middle: Representative flow cytometric analysis showing the percentage of GC B cells gated on splenic B cells in mice 7 days post-immunization with sheep red blood cells (SRBCs). Right: Total number per spleen of GC B cells 5, 7, 12 or 15 days post-SRBC immunization. **(F)**. *Rag1^KO/KO^ Card11^+/+^* mice were irradiated and reconstituted with *Ptprc^a/a^ Card11^+/+^* (black fill) bone marrow in a 1:1 mixture with *Ptprc^b/b^ Card11^+/+^* (grey fill) or *Card11^M365K/M365K^* (red fill) bone marrow, and sacrificed 7 days post-SRBC immunization. Graphs show B cells as a percentage of *Ptprc^a/a^* or *Ptprc^b/b^* splenic leukocytes, or GC B cells as a percentage of *Ptprc^a/a^* or *Ptprc^b/b^* B cells, in mice that received bone marrow of the indicated genotypes. **(C-E)**. Statistical comparisons made by *t*-test, corrected for multiple comparisons using the Holm-Sidak method. Data are representative of *n* > 2 independent experiments with *n* > 5 mice per group. **(E)**. Data are pooled from 3 independent experiments. Comparison made after excluding the one high outlier. **(F)**. Statistical comparisons made by paired *t*-test. not significant (n.s) p > 0.05; * *p* < 0.05; ** *p* < 0.01; *** *p* < 0.001.

Here, we addressed these open questions by analyzing B and T lymphocytes in mice with a germline *Card11^M365K^* mutation identical to *CARD11^M365K^* previously identified in DLBCL (30) and modifying a conserved region of the CC domain recurrently mutated in B-lymphomas (31) and T-lymphomas (38, 39, 44–46) (**Figure 1A**). CARD11.M365K increased activation and proliferation of B and T lymphocytes following antigen-receptor stimulation. *Card11^M365K^* mice had increased numbers of GC B cells before and at multiple timepoints during a T cell-dependent response to immunization. *Card11^M365K^* mutation was insufficient to cause lymphoma, or B cell lymphocytosis as observed in individuals with BENTA disease. However, *Card11^M365K^* mutation caused mutant allele dose-dependent, cell-autonomous accumulation of T follicular (T_FH_), T regulatory (T_REG_) and T follicular regulatory (T_FR_) cells over-expressing stimulatory and inhibitory checkpoint molecules. Our findings add to our understanding of CARD11 as a critical signaling protein in lymphocytes. They reveal T_FH_, T_REG_ and T_FR_ cells as T cell populations particularly sensitive to CARD11 signaling, and help to explain the recurrence of somatic GOF *CARD11* mutations in aggressive human T-lymphomas arising from CD4, T_REG_ and T_FH_ cells.

## 2 Materials and methods

### 2.1 Mice

All animals care, housing and experiments were performed in accordance with approved protocols of: (1) the ANU National University Animal Experimentation Ethics Committee, for mice on a C57BL/6 NCrl background; (2) the Garvan Institute of Medical Research/St Vincent’s Hospital Animal Ethics Committee, for mice on a C57BL/6 JAusb background. All experiments conformed to the current guidelines from the Australian Code of Practice for the Care and Use of Animals for Scientific Purposes. Within independent experiments, *Card11* wild-type and mutant animals were sex- and age-matched.

*Card11^M365K^* mice harbor a germline A to T nucleotide substitution at position 140,889,709 on chromosome 5, resulting in a methionine to lysine M365K substitution in the highly conserved region of the coiled-coil domain of CARD11. *Card11^M365K^* mice were obtained by exome sequencing of first-generation offspring of C57BL/6 mice exposed to N-ethyl-N-nitrosourea (ENU; databases.apf.edu.au/mutations) and bred to homozygosity on a C57BL/6 Ncrl background. *Card11^M365K^* mice were rederived onto a C57BL/6 JAusb background upon transfer from the Australian National University (ANU) Australian Phenomics Facility (APF) to Australian BioResources (ABR; MossVale, Australia).

*Card11^loco^* mice harbor 3 distinct single-nucleotide variants in *Card11* introns 2, 10 and 20 that cause a complete loss of CARD11 protein expression (56). *Card11^loco^* mice were also identified by exome sequencing of first-generation offspring of C57BL/6 mice exposed to ENU (databases.apf.edu.au/mutations). The mice were bred to homozygosity and maintained on a C57BL/6 NCrl background.

C57BL/6 NCrl, C57BL/6 JAusb, B6.JSL-*Ptprc^a^Pepc^b^* (CD45.1) and B6.129S7-*Rag1^tm1Mom^*/J (*Rag1^KO/KO^*) mice were purchased from ABR.

### 2.2 Flow cytometry

Single-cell suspensions were prepared from mouse spleen, bone marrow, inguinal lymph nodes, peritoneal cavity and blood. 1-4 x 10^6^ cells in PBS 2% FCS were transferred into appropriate wells of a 96-well U bottom plate. To prevent non-specific antibody binding, cells were incubated with F_c_ blocking antibody for 20 min at 4°C in the dark. Cells were then incubated with antibodies for 30 min, on ice and in the dark. To fix cells, they were incubated in 10% formalin (Sigma-Aldrich) for 15 min at 4°C, and washed and resuspended in PBS 2% FCS. To stain for intracellular nuclear proteins, cells were fixed and permeabilised using the manufacturer’s instructions and the eBioscience Transcription Factor Staining kit. Stained single-cell suspensions were acquired on the BD LSRFortessa™.

Where appropriate, following extracellular antibody staining, immune populations were sorted by fluorescence-activated cell sorting (FACS) on a FACS Aria III (BD Biosciences).

### 2.3 Antibodies used for flow cytometry

Antibodies used for flow cytometric study of mouse organs are listed in **Table 1**.

**Table 1:** Antibodies used for flow cytometric analyses of mouse hematopoietic cells.

Cat. number	Antibody	Fluorochrome	Company	Clone
122007	CD28	FITC	BioLegend	E18
100204	CD3	FITC	BioLegend	17A2
100217	CD3	PerCP Cy5.5	BioLegend	17A2
563331	CD4	BV786	BD Biosciences	GK1.5
564306	CD4	BUV737	BD Biosciences	SK3
17-0051-81	CD5	APC	Thermo Fischer	53-7.3
563796	CD8	BUV395	BD Biosciences	53-6.7
557654	CD8	APC Cy7	BD Biosciences	53-6.7
45-0114-82	CD11c	PerCP/Cy5.5	eBioscience	N418
115546	CD19	BV510	BioLegend	6D5
553818	CD21/35	FITC	BD Biosciences	7G6
101614	CD23	PE/Cy7	BioLegend	B3B4
101820	CD24	Pacific Blue	BD Biosciences	M1/69
102030	CD25	PerCP Cy5.5	BioLegend	PC61
558642	CD25	PE	BD Biosciences	7D4
557192	CD25	APC	BD Biosciences	PC61
562768	CD38	BV421	BD Biosciences	Ab90
553270	CD43	FITC	BD Biosciences	S7
563058	CD44	BV605	BD Biosciences	IM7
103020	CD44	Pacific Blue	BioLegend	IM7
553133	CD44	FITC	BD Biosciences	IM7
564449	CD45R/B220	BUV737	BD Biosciences	RA3-6B2
104438	CD62L	BV605	BioLegend	MEL-14
560513	CD62L	PerCP Cy5.5	BD Biosciences	MEL-14
104508	CD69	PE	BioLegend	H1.2F3
740877	CD86	BV786	BD Biosciences	GL1
17-5892-83	CD93	APC	eBioscience	AA4.1
17-1522-80	CD152 (CTLA-4)	APC	Thermo Fischer	UC10-4B9
12-9949-81	CD278 (ICOS)	PE	Thermo Fischer	C398.4A
313529	CD278 (ICOS)	APC/Cy7	BioLegend	C398.4A
25-9985-82	CD279 (PD1)	PE Cy7	Thermo Fischer	J43
551892	CD279 (PD1)	PE	BD Biosciences	J43
551961	CXCR5	Purified	BD Biosciences	2G8
551960	CXCR5	Biotin	BD Biosciences	2G8
126406	FoxP3	AF488	BioLegend	MF-14
25-5773-82	FoxP3	PE/Cy7	Thermo Fischer	FJK-16s
48-5773-80	FoxP3	eF450	Thermo Fischer	FJK-16s
565988	IgD	BUV395	BD Biosciences	11-26c.2a
559750	Ig, κ light chain	Biotin	BD Biosciences	187.1
407308	Ig, λ light chain	PE	BioLegend	RML-42
406515	IgM	APC/Cy7	BioLegend	RMM-1
405229	Streptavidin	BV605	BioLegend	N/A
109220	TCRb	APC Cy7	BioLegend	H57-597
109227	TCRb	PerCP Cy5.5	BioLegend	H57-597

### 2.4 Retroviral gene transfer system

To evaluate the effect of the CARD11.M365K substitution, we used a retrovirus gene transfer and culture system to introduce into primary activated B cells the following: CARD11.M365K or as controls wild-type CARD11, BENTA-associated (27–29) CARD11.G123S, CARD11.E134G or empty vector expressing EGFP only.

Briefly, replication-defective retrovirus particles were produced by the Pheonix ecotropic helper-free retrovirus packaging cell line (ATCC; CRL-3214), and transduction efficiency measured by flow cytometric measurement of EGFP expression. C57BL/6 B cells were stimulated with 10µg/mL goat anti-mouse IgM (Jackson ImmunoResearch) and 10µg/mL anti-CD40 (FGK4.5; BioXCell) for 24 hours, followed by spin-infection with retrovirus supernatant containing DOTAP (Roche). The cells were then cultured in fresh RPMI 10µg/mL anti-CD40 for 36 hours, washed with RPMI and resuspended in cRPMI at a density of 10^6^ cells/mL.

The number of live EGFP^+^ cells was determined by hemocytometer counting of trypan blue–negative cells in each culture, and flow cytometric analysis of the same cells.

### 2.5 T cell proliferation assays

Approximately 20 x 10^6^ total splenocytes were incubated for 5 min at room temperature in 1 mL RPMI-1640 (Gibco) containing Cell Trace Violet (CTV; Invitrogen) at a final concentration of 20 μM, followed by three washes in complete RPMI (RPMI-1640 containing 10% heat-inactivated fetal calf serum (HI-FCS), 2% Penicillin-Streptomycin-Glutamine (Gibco), 0.1% 50 mM 2-Mercaptoethanol). CTV-labelled splenocytes were plated at a density of 1 x 10^6^ cells per mL and incubated for 3 to 5 days in complete RPMI alone or containing 10 μg/mL anti-CD3 and 10 μg/mL anti-CD28. Cell divisions were enumerated by flow cytometric measurements of the fluorescence intensity of CTV.

### 2.6 *In vitro* analysis of CARD11^M365K^ and CARD11^G123S^ function

CARD11 mutations M365K and G123S were introduced into the corresponding mouse *Card11* sequence using PCR-based site-directed mutagenesis. The coding sequences for *Card11* and its variants were fused with the mutant ecDHFR sequence (kindly provided by Dr Wandless, Stanford university) in mammalian expression vector pcDNA3.1+ (66). HEK293 cells were transfected with expression vectors for ecDHFR-CARD11 mutants and reporter plasmids expressing firefly luciferase and Renilla luciferase under NF-kB and thymidine kinase promoters, respectively (pGL4.32 and pGL4.74 from Promega). The expression of CARD11 variants was induced by the addition of 10 mM trimethoprim (TMP). The transfected cells were lysed 5 hr after TMP addition, and luciferase activity was measured by Dual-Luciferase Reporter Assay (Promega).

### 2.7 Th differentiation assay

Sorted *Card11^M365K^* mutant or wild-type naïve CD4 T cells were sorted to high purity by FACS, and cultured in flat bottom 96-well plates coated with 4 μg/mL anti-CD3 (BioLegend), in RPMI1640 (Life technologies) supplemented with 10% heat inactivated FCS (Life technologies), 5×10^-5^ M 2-ME, 0.1mM non-essential amino acids, 1mM sodium pyruvate, 10mM HEPES, 100u/mL penicillin, 100ug/mL Streptomycin, 100ug/mL Noromycin (all from Sigma) at a density of 0.5 ×10^6^ cells/mL.

The naïve CD4 T cells were cultured for 4 days in the following polarizing conditions: Th0 (1 μg/mL anti-CD28, 5 μg/mL anti-TGFβ, 5 μg/mL anti-IL-4, 5 μg/mL anti-IFNγ); Th1 (1 μg/mL anti-CD28, 5 μg/mL anti-TGFβ, 5 μg/mL anti-IL-4, 10ng/mL IL-12); Th2 (10ng/mL IL-4, 1 μg/mL anti-CD28, 5 μg/mL anti-TGFβ, 5 μg/mL anti-IFNγ); Th17 (20ng/mL IL-6, 1ng/mL human TGFβ, 5 μg/mL anti-IFNγ, 5 μg/mL anti-IL-4, 1 μg/mL anti-CD28).

After 4 days of culture, cells were stimulated with PMA (50ng/mL) and ionomycin (375ng/mL) for 6 hrs. Brefeldin A (10 μg/mL) was added to each well after 2 hours of stimulation. Cells were harvested, washed and stained with Zombie Aqua Viability dye (BioLegend), fixed with 2% formalin, permeabilized with saponin (0.1%), and stained intracellularly with mAbs directed against TNFα, IFNγ, IL17A, IL-2, IL5, IL-4.

### 2.8 Mixed bone marrow chimeras

Age- and sex-matched *Card11^+/+^ Rag1^KO/KO^* C57BL/6J recipient mice were irradiated with one dose of 425 Rad from an X-ray source (X-RAD 320 Biological Irradiator, PXI). Recipient mice were then intravenously injected with 4 x 10^6^ bone marrow cells consisting of a 1:1 mixture of *Card11^+/+^* C57BL6 CD45.1^+^ (*Ptprc^a/a^*) bone marrow cells and CD45.2^+^ (*Ptprc^b/b^*) bone marrow cells that were *Card11^+/+^* or *Card1^M365K/M365K^*. 7 weeks were allowed for immune reconstitution before intravenous immunization of recipient mice with 2 x 10^8^ SRBCs. The immunized chimeric mice were sacrificed 7 days post-immunization.

### 2.9 CD4 T cell adoptive transfer and anti-PD-1 treatment

8-12 weeks old *Card11^M365K^* mice were sacrificed and single-cell suspensions prepared from their spleens. Splenic CD4 T cells were isolated by incubation with anti-CD4 biotin antibody and positive enrichment by manual magnetic-activated cell sorting (MACS) using LS columns (Miltenyi Biotec). 3-4 x 10^6^ *Card11^+/+^* or *Card11^M365K/M365K^* CD4 T cells were intravenously transferred into each recipient mouse: either into C57BL6.CD45.1^+^ recipients where donor cells could be isolated based on CD45.1/2 expression, or in an independent experiment into *Rag1^KO/KO^* mice that lack mature B and T cells (67). Recipient mice were treated with intraperitoneal *(i.p.)* injection of 200 μg anti-mouse PD-1 (clone RMP1-14; BioXCell) or rat IgG2a anti-trinitrophenol isotype control (clone 2A3; BioXCell) at days 0, 2 and 5 post-CD4 T cell transfer. Recipient mice were sacrificed 7 days post-injection, and blood and spleen harvested for analysis.

### 2.10 Statistical analysis

Statistical analysis of flow cytometric experiments was performed using the GraphPad Prism 6 software (GraphPad, San Diego, USA). A one-tailed unpaired Student’s t-test with Welch’s correction was used for comparisons between two normally distributed groups. An unpaired student’s t-test, corrected for multiple comparisons using the Holm-Sidak method, was used for comparisons of more than two groups. Differences between paired measurements were analyzed by paired *t*-test. In all graphs presented, the error bars represent the mean and standard deviation. * p < 0.05, ** p < 0.01, *** p < 0.001.

## 3 Results

### 3.1 CARD11.M365K is a GOF protein that increases BCR-induced activation and proliferation *in vitro*

We identified the novel *Card11^M365K^* mouse strain by exome sequencing of first-generation offspring of C57BL/6 mice exposed to the mutagen N-ethyl-N-Nitrosourea (ENU). *Card11^M365K^* mutant mice carry an A to T nucleotide substitution at position 140,889,709 on Chromosome 5, resulting in a methionine to lysine change at amino acid 365 (**Figure 1A**).

To determine the effects in mouse B cells of *Card11^M365K^* mutation relative to known GOF *Card11* mutations, we used a retroviral gene transfer and culture system to transduce *Card11^M365K^* into primary activated B cells (**Supplementary Figure 1**). As controls, B cells were otherwise transduced with an empty vector expressing EGFP only, expressing wild-type *Card11* or *Card11^G123S^*, found in patients with BENTA disease, DLBCL and ATL (27–29, 31), or expressing *Card11^E134G^* found in several patients with BENTA disease (27–29). *Card11* ^M365K^-transduced B cells expressed lower B220 and higher CD86 (**Supplementary Figure 1A**) cell-surface levels compared to control vector-transduced B cells, indicative of increased NF-κB activation in these cells. *Card11^M365K^*-transduced B cells expressed CD86 and B220 at levels intermediate between *Card11^E134G^*- and *Card11^G123S^*-transduced cells, and over a period of four days in culture, *Card11^M365K^*-transduced B cells accumulated in numbers intermediate between *Card11^E134G^*- and *Card11^G123S^*-transduced cells (**Supplementary Figure 1B**). *CARD11^M365K^* was previously shown to enhance NF-κB activity in a luciferase assay (30). To validate this and directly measure the effects of M365K and G123S mutations on NF-κB signalling, we utilized our previously published (65) luciferase reporter method. Following trimethoprim-induced expression, M365K and G123S mutant CARD11 caused a mean 5-fold and 11-fold higher induction of the NF-κB luciferase reporter, respectively, relative to that induced by wild-type CARD11 (**Supplementary Figure 1C**). Collectively, these results indicate that M365K mutation causes CARD11 GOF intermediate between that caused by E134G or G123S.

To test the effects on B cells of *Card11^M365K^* mutation within an otherwise normal gene, we measured survival and proliferation of splenic B cells from *Card11^M365K/+^* relative to *Card11^+/+^* mice. As an additional control, we included splenic B cells from homozygous *Card11^loco/loco^* mice harboring 3 distinct single-nucleotide variants that cause a complete loss of CARD11 protein expression (68). Over a period of 5 days in the absence of stimulation, the percentage of live *Card11^+/+^* versus *Card11^M365K/+^* B-lymphocytes decreased at the same rate, whilst live *Card11^loco/loco^* B cells decreased in frequency more rapidly (**Supplementary Figure 1D**). CARD11.M365K therefore does not enhance B cell survival in absence of stimulation. Similar results were obtained following stimulation with a 1 μg/mL sub-mitogenic dose of anti-IgM (**Supplementary Figure 1E**).

To measure proliferation following stimulation, we labelled splenic B cells with Cell Trace Violet (CTV). Relative to Card11^+/+^ B cells, Card11^M365K/+^ cells increased in size faster and Card11^loco/loco^ cells more slowly, following stimulation with different concentrations of anti-IgM (**Supplementary Figure 1E**). B cells stimulated with 10 μg/mL anti-IgM divided up to 5 times and a mildly increased percentage of Card11^M365K/M365K^ B cells divided 3 or more times relative to Card11^+/+^ cells, whereas 80% of Card11^loco/loco^ B cells failed to divide at all (**Supplementary Figure 1F**). The mean percentage of divided cells was 70% for Card11^M365K/M365K^, 62% for Card11^M365K/+^ and 57% for Card11^+/+^ B cells. By contrast, only 27% of Card11^loco/loco^ B cells had divided (**Supplementary Figure 1G**). Given the small number of WT CD4 T cells assessed, we were unable to conclude that these effects were statistically significant.

CARD11.M365K is thus a mild GOF protein that increases BCR-stimulated activation, survival and to a small extent proliferation.

### 3.2 Germline *Card11^M365K^* mutation causes accumulation of germinal center B cells

To determine the effects of *Card11^M365K^* mutation *in vivo*, we analyzed *Card11^M365K^* mice on a C57BL/6 JAusb or C57BL/6 Ncrl background. All results presented herein were consistent between backgrounds and unless specified otherwise, all figures present data from C57BL/6 JAusb mice. Following inter-cross of heterozygous mutant mice, *Card11^M365K/+^* and *Card11^M365K/M365K^* mice were detected at expected Mendelian frequencies at time of weaning and genotyping (**Figure 1B**). Heterozygous and homozygous mutant mice developed no obvious pathologies and had comparable weight and survival to wild-type mice over a period of up to 50 weeks (**Figure 1B**). Germline *Card11^M365K^* mutation therefore appears insufficient to cause overt pathology in mice.

Given the recurrence of somatic *CARD11* GOF mutations in B lymphomas (31), and the effects of germline *CARD11* GOF mutations on B cells in mice and humans (22–29, 52–54), we assessed B cell populations in the bone marrow, spleen and lymph nodes of wild-type and *Card11^M365K^* mutant mice. *Card11^+/+^*, *Card11^M365K/+^* and *Card11^M365K/M365K^* mice had comparable percentages of Lin^neg^ Sca-1^pos^ c-Kit^pos^ (LSK) stem cells in the bone marrow (**Supplementary Figure 2A**). 5-7 week-old *Card11^M365K^* mutant mice had a reduced percentage of CD5^pos^ CD11b^pos^ CD23^low^ CD43^high^ peritoneal cavity B1a cells. Interestingly, this difference waned with age (**Supplementary Figure 2B**). *Card11^M365K^* mutant mice also had comparable percentages of bone marrow leukocytes of the B220^pos^ B-lineage, and within these of IgM^neg^ IgD^neg^ precursor, IgM^pos^ IgD^int^ immature or IgM^low^ IgD^pos^ mature recirculating B cells (**Supplementary Figure 2C**), and comparable percentages of precursor B cells with a CD43^high^ CD24^neg^ pre-pro-, CD43^int^ CD24^int^ pro- or CD43^low^ CD24^pos^ pre-B phenotype (**Supplementary Figure 2C**).

Notably, *Card11^M365K^* mutant mice had increased cellularity and increased percentage of B leukocytes in the spleen and inguinal lymph nodes (**Figure 1C**, **Supplementary Figure 2D**). *Card11^M365K^* mutant mice had normal numbers of CD93^+^ transitional and CD93^-^ mature B cell populations (**Supplementary Figure 2E**), but though unimmunized, had an increased percentage and number of germinal center (GC) B cells in both spleen and lymph nodes (**Figure 1D**). We therefore studied the effect of *Card11^M365K^* mutation on T cell-dependent GC responses, by immunizing *Card11^M365K^* mice with sheep red blood cells (SRBCs) and sacrificing them 5, 7, 12 or 15 days later. Relative to wild-type controls, *Card11^M365K/M365K^* mice had increased numbers of B220^pos^ CD38^low^ CD95^pos^ GC B cells at days 7, 12 and 15 post-immunization (**Figure 1E**).

To test whether CARD11.M365K drives GC B cell accumulation cell-autonomously or rather secondary to dysregulation of T cells or other hematopoietic cells, we generated mixed chimeras wherein a fraction of all hematopoietic cells had mutant *Card11^M365K/M365K^* and the remainder had wild-type *Card11*. *Card11^+/+^ Rag1^KO/KO^* mice were irradiated and transplanted with an equal mixture of *Card11^M365K/M365K^ Ptprc^b/b^* and control *Card11^+/+^ Ptprc^a/a^* bone marrow. As an additional control, another set of mixed chimeras received an equal mixture of *Card11^+/+^ Ptprc^a/b^* and *Card11^+/+^ Ptprc^a/a^* bone marrow. All chimeras were immunized with sheep red blood cells (SRBCs) and sacrificed 7 days later. Flow cytometric analysis revealed no significant difference in frequency of B cells or of germinal center B cells of *Card11^+/+^* versus *Card11^M365K/M365K^* donor origin (**Figure 1F**). *Card11^M365K/M365K^* thus provides no striking cell-autonomous advantage to GC B cells, 7 days post-SRBC immunization in this model.

### 3.3 Germline *Card11^M365K^* mutation causes accumulation of activated CD8 and CD4 T cells, T_FH_, T_FR_ and T_REG_ cells

**Figure 2:**
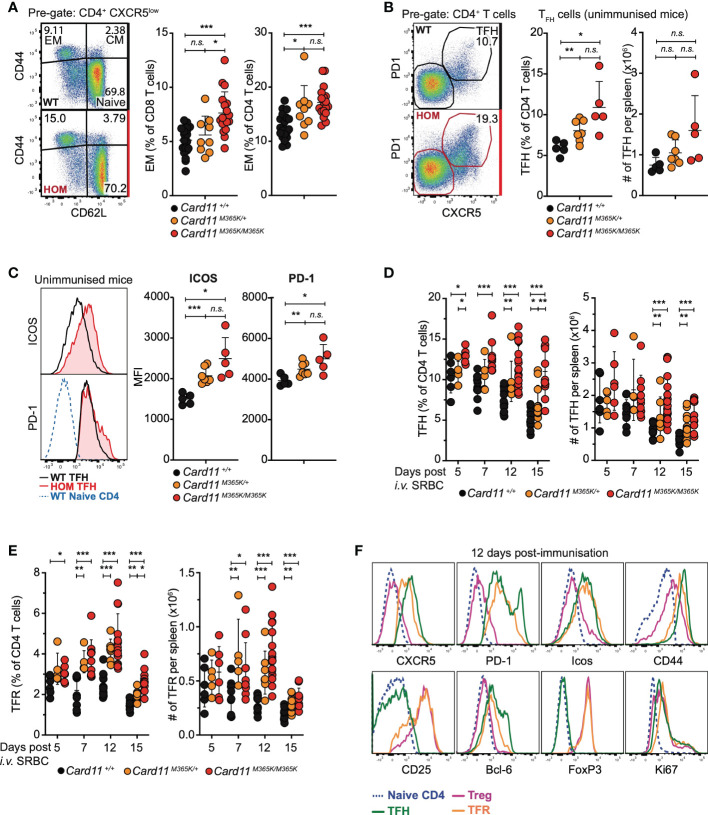
Germline GOF *Card11^M365K^* mutation causes accumulation of effector CD4 and CD8 T cells, T follicular and T follicular regulatory T cells. **(A-C)**. Flow cytometric analysis of T cell populations in non-immunized mice of the indicated genotypes. **(A)**. Left, representative flow cytometric analysis and right, percentage of CD44^-^ CD62L^+^ naïve, CD44^+^ CD62L^+^ central memory (CM), and CD44^+^ CD62L^-^ effector memory (EM) subsets among splenic CD8 or CD4 T cells. **(B)**. Left, representative flow cytometric analysis and right, percentage among CD4 T cells or total number per spleen of CXCR5^high^ PD-1^high^ follicular helper (T_FH_)-like CD4 T cells. **(C)**. Left, representative flow cytometric histograms showing distribution of ICOS (top) or PD-1 (bottom) fluorescence on *Card11^+/+^* naïve CD4 T cells, *Card11^+/+^* or *Card11^M365K/M365K^* T_FH_ -like cells. Right, plots showing mean fluorescence intensity (MFI) of ICOS or PD-1 on T_FH_-like cells from mice of the indicated genotypes. **(D-F)**. Flow cytometric analysis of T cell populations in mice 5, 7, 12 or 15 days post-immunization with sheep red blood cells (SRBCs). **(D)**. FoxP3^-^ CXCR5^high^ PD-1^high^ T_FH_ cells as a percentage of CD4^+^ TCRβ^+^ T cells or as total number per spleen, in mice of the indicated genotypes. **(E)**. CXCR5^high^ PD-1^high^ CD25^+^ FoxP3^+^ T follicular regulatory (T_FR_) cells as a percentage of CD4^+^ TCRβ^+^ T cells or as total number per spleen, in mice of the indicated genotypes. **(F)**. Representative flow cytometric histograms showing distribution of cell-surface CXCR5, PD-1, ICOS, CD44, CD25 and intracellular Bcl-6, FoxP3, Ki-67 fluorescence in naïve CD4 (blue), T_FH_ (green), T regulatory (T_REG_; magenta) or T_FR_ (orange) cells from *Card11^M365K/M365K^* mice 12 days post-SRBC immunization. The histograms are also representative of results from *Card11^+/+^* mice and from results 7 and 15 days post-immunization. **(A-E)**. Statistical comparisons made by *t*-test, corrected for multiple comparisons using the Holm-Sidak method. Data are representative of *n* > 2 independent experiments with *n* > 4 mice per group. not significant (n.s) p > 0.05; * *p* < 0.05; ** *p* < 0.01; *** *p* < 0.001. **(A, D, E)**. Data are pooled from 3 independent experiments.

Based on the recurrence of somatic *CARD11* GOF mutations in PTCL (38, 39, 43–46), we hypothesized that *Card11^M365K^* mutation would dysregulate T cells. Following flow cytometric analysis of T cell populations, we observed a mutant allele gene dose-dependent increase in percentage (but not total number) of CD62L^-^ CD44^+^ effector memory CD8 and CD4 T cells in the spleen and lymph nodes of *Card11^M365K^* mutant relative to wild-type mice (**Figure 2A**). The increased fraction of effector memory CD8 T cells was not accompanied by changes in their surface expression of CX3CR1, KLRG1, NKG2D or by changes in their granularity, whereas by contrast an increased fraction of *Card11*-mutant effector memory CD8 T cells expressed high levels of CD69 (**Supplementary Figure 3A**). We observed no change in fraction of CD8 T cells expressing the cytotoxic effector molecule granzyme B (**Supplementary Figure 3B**). Unimmunized *Card11^M365K/+^* and to a greater extent *Card11^M365K/M365K^* mice had an accumulation of TCRβ^+^ CD3^+^ CD4^+^ CXCR5^high^ PD-1^high^ T follicular helper (T_FH_)-like cells (**Figure 2B**). These accumulating *Card11*-mutant T_FH_-like cells expressed homogenously higher cell-surface levels of ICOS and some but not all expressed higher cell-surface levels of PD-1, relative to wild-type cells (**Figure 2C**). To test whether germline *Card11^M365K^* mutation also increases accumulation of T_FH_ cells during T cell-dependent responses, we analyzed the same mice described earlier at 5, 7, 12 and 15 days post-immunization with SRBCs. *Card11^M365K/M365K^* mice had an increased frequency and total number of splenic T_FH_ cells at days 7, 12 and 15 (**Figure 2D**). Interestingly, they also had a significant accumulation of TCRβ^+^ CD3^+^ CD4^+^ CXCR5^+^ PD-1^+^ CD25^+^ FoxP3^+^ T follicular regulatory (T_FR_) cells (**Figure 2E**). As expected, the T_FH_ cells were Bcl-6^high^, ICOS^high^ and CD44^+^ and the T_FR_ cells were FoxP3^+^, Bcl-6^+^, ICOS^high^, CD44^+^ and Blimp-1^high^ (**Figure 2F**). Similar to our observations in unimmunized mice, the accumulating *Card11^M365K/M365K^* T_FH_ and T_FR_ cells expressed homogeneously higher levels of ICOS relative to their *Card11^+/+^* counterparts.

**Figure 3:**
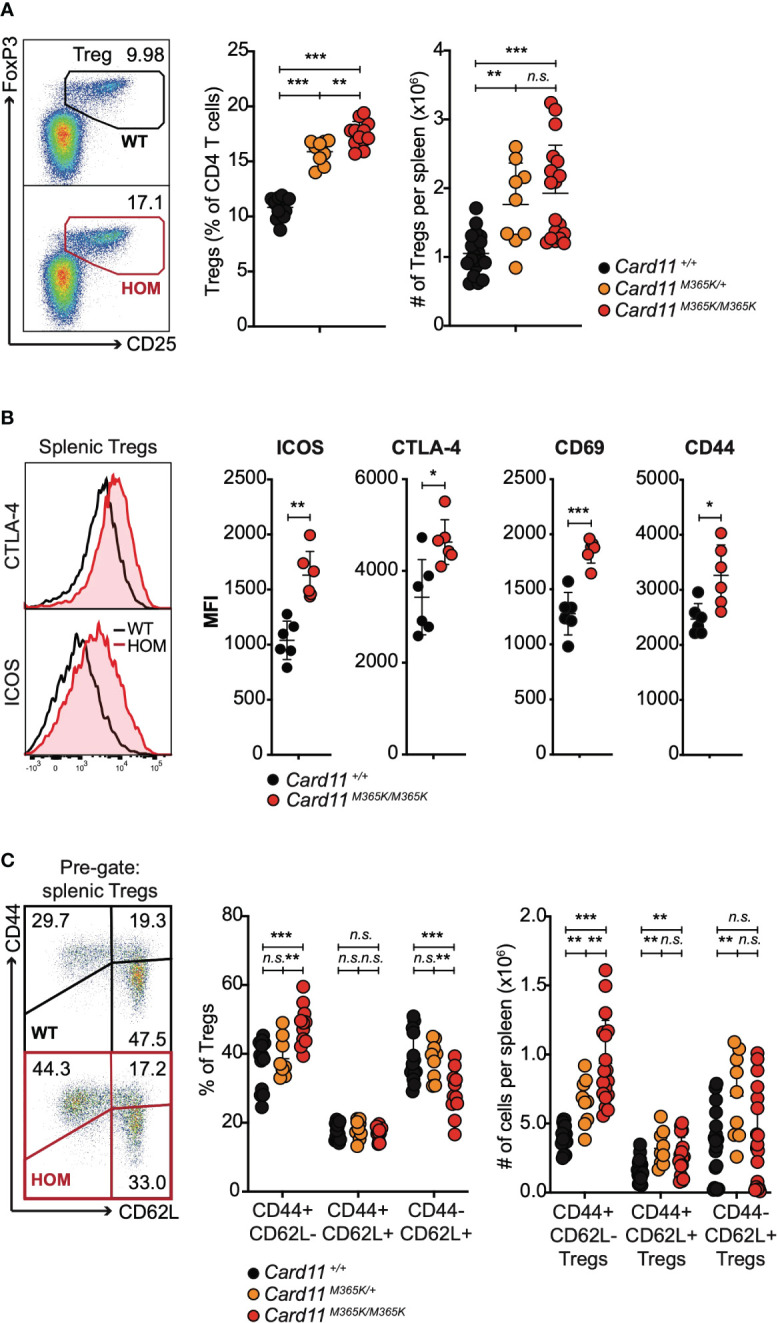
Germline GOF *Card11^M365K^* mutation causes accumulation of CD62L^neg^ CD44^high^ ICOS^high^ CTLA-4^high^ T regulatory cells. **(A)**. Left, representative flow cytometric analysis and right, percentage amongst CD4 T cells or total number per spleen of TCRβ^+^ CD4^+^ CD25^+^ FoxP3^+^ T regulatory (T_REG_) cells, in mice of the indicated genotypes. **(B)**. Left, representative flow cytometric histograms showing distribution of intracellular CTLA-4 and cell-surface ICOS fluorescence and right, plots of mean fluorescence intensity (MFI) of ICOS, CTLA-4, CD69 or CD44, for splenic T_REGS_ from mice of the indicated genotypes. **(C)** Left, representative flow cytometric analysis and right, percentage amongst T_REGS_ or total number per spleen of CD44^-^ CD62L^+^, CD44^+^ CD62L^+^ and CD44^+^ CD62L^-^ T_REGS_, in mice of the indicated genotypes. **(A-C)**. Statistical comparisons made by *t*-test, corrected for multiple comparisons using the Holm-Sidak method. Data are representative of *n* > 2 independent experiments with *n* > 4 mice per group. not significant (n.s) p > 0.05; * *p* < 0.05; ** *p* < 0.01; *** *p* < 0.001. **(A, C)** Data are pooled from 3 independent experiments.

Unimmunized *Card11^M365K/+^* and to a greater extent *Card11^M365K/M365K^* mice had a mutant allele dose-dependent increase accumulation of T_REG_ cells (**Figure 3A**), of phenotype TCRβ^+^ CD3^+^ CD4^+^ CD25^+^ FoxP3^+^ and having first excluded CXCR5^high^ PD-1^high^ T_FH_-like or T_FR_-like cells. The accumulating *Card11*-mutant T_REGS_ expressed homogeneously higher levels of ICOS but also of CTLA-4, and higher levels of CD69 and CD44 (**Figure 3B**), and the *Card11*-mutant mice had a significant accumulation of T_REG_ cells with a CD62L^-^ CD44^+^ effector memory-like phenotype (**Figure 3C**). Similarly, *Card11^M365K^* mice on a C57BL/6 Ncrl background had a significant increase in percentage and total number per spleen of CD44^high^ and PD-1^high^ CD4 and CD8 T cells (**Supplementary Figures 3A-C**) and of T_FH_-like and T_REG_ cells, which were by contrast significantly reduced in *Card11^loco/loco^* mice (**Supplementary Figures 3D, E**).

**Figure 4:**
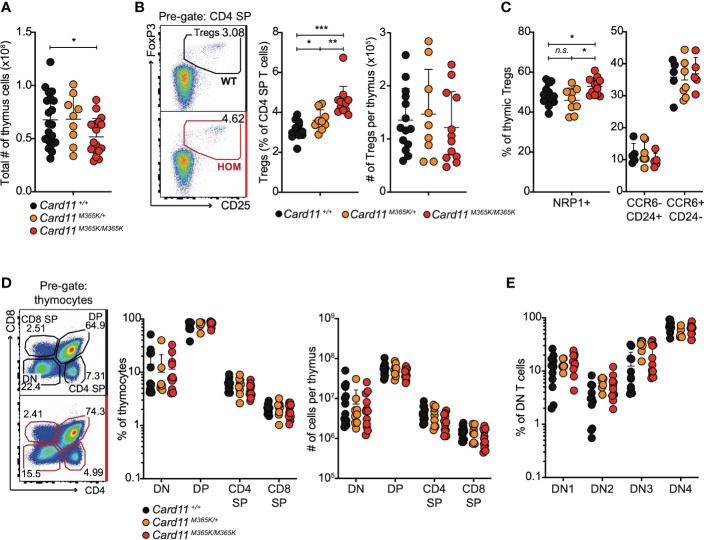
*Card11^M365K^* mutant mice have normal numbers of thymic T cell precursors, single positive CD4 and CD8 T cells and T regulatory cells. **(A)**. Total number of cells per thymus from individual mice of the indicated genotypes. **(B)**. Left, representative flow cytometric analysis and right, percentage amongst CD4 single-positive (SP) T cells or total number per thymus of CD25^+^ FoxP3^+^ CD4^+^ T regulatory (T_REG_) cells, in mice of the indicated genotypes. **(C)**. Percentage of NRP1^+^ thymic T_REGS_ (left) or of CCR6^-^ CD24^+^ or CCR6^+^ CD24^-^ thymic T_REGS_ (right), in mice of the indicated genotypes. **(D)**. Left, representative flow cytometric analysis and right, percentage amongst thymocytes or total number per thymus of CD4^-^ CD8^-^ double-negative (DN), CD4^+^ CD8^+^ double-positive (DP), CD4^+^ SP or CD8^+^ SP T cells, in mice of the indicated genotypes. **(E)**. Percentage of DN T cells with a CD25^-^ CD44^+^ DN1, CD25^+^ CD44^+^ DN2, CD25^+^ CD44^-^ DN3 or CD25^-^ CD44^-^ DN4 phenotype, in mice of the indicated genotypes. Statistical comparisons made by *t*-test, corrected for multiple comparisons using the Holm-Sidak method. Data are representative of *n* > 2 independent experiments with *n* > 4 mice per group. not significant (n.s) p > 0.05; * *p* < 0.05; ** *p* < 0.01; *** *p* < 0.001. **(A, B)** Data are pooled from 3 independent experiments.

Given the above findings, we tested whether germline *Card11^M365K^* mutation alters early T cell development in the thymus. Thymus cellularity was similar in *Card11^M365K/+^* but mildly decreased in *Card11^M365K/M365K^* relative to *Card11^+/+^* mice (**Figure 4A**). *Card11*-mutant mice had a significantly increased percentage of CD25^+^ FoxP3^+^ T_REGS_ among CD4 single-positive (SP) cells, but no change in total number of thymic T_REGS_, relative to wild-type mice (**Figure 4B**). Cell-surface Neuropilin-1 (NRP1), CCR6 and CD24 were used to identify peripherally induced versus newly developed or recirculating thymus-derived T_REGS_ (68–70). We observed no change in percentage (or total number) of thymus-derived NRP1^+^, thymus-derived nascent CCR6^-^ CD24^+^ or recirculating CCR6^+^ CD24^-^ T_REGS_ in *Card11*-mutant relative to wildtype mice (**Figure 4C**). *Card11* wild-type and mutant mice also had comparable frequencies and numbers of CD4^-^ CD8^-^ double-negative (DN), CD4^+^ CD8^+^ double-positive (DP), CD4^+^ single-positive (SP) and CD8^+^ SP thymocytes (**Figure 4D**), and of CD44^+^ CD25^-^ DN1, CD44^+^ CD25^+^ DN2, CD44^-^ CD25^+^ DN3 and CD44^-^ CD25^-^ DN4 early progenitors (**Figure 4E**). Thymic T cell development thus appears overtly normal in *Card11^M365K^* mutant mice.

Collectively, the above results demonstrate that germline CARD11 gain-of-function causes over-accumulation in the periphery of activated CD8 and CD4 T cells, T_FH_, T_FR_ and T_REG_ cells expressing increased levels of activating and inhibitory checkpoint molecules.

### 3.4 *Card11^M365K^* mutation provides a cell-autonomous advantage to activated CD8 and CD4 T cells, to T_FH_, T_REG_ and T_FR_ cells

**Figure 5:**
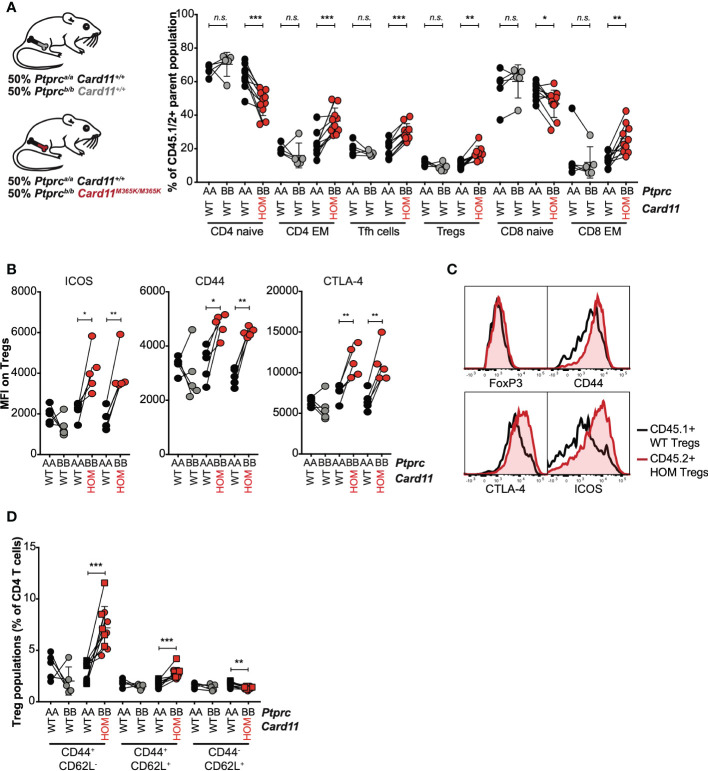
GOF *Card11^M365K/M365K^* mutation provides a cell-intrinsic advantage to activated CD8 and CD4 T cells, T follicular helper-like and T regulatory cells. **(A-D)**. Mixed chimeras were generated by irradiating *Rag1^KO/KO^ Card11^+/+^* mice and reconstituting them with *Ptprc^a/a^ Card11^+/+^* (black fill) bone marrow in a 1:1 mixture with *Ptprc^b/b^ Card11^+/+^* (grey fill; 1 donor) or *Card11^M365K/M365K^* (red fill; 2 separate donors) bone marrow. These mixed chimeras were sacrificed 7 days post-immunization with SRBCs. **(A)**. Percentage, within the corresponding *Ptprc^a/a^* or *Ptprc^b/b^* parent population, of CD44^-^ CD62L^+^ naïve and CD44^+^ CD62L^-^ effector memory (EM) CD4 T cells, CXCR5^high^ PD-1^high^ T follicular helper (T_FH_) and CD25^+^ FoxP3^+^ T regulatory (T_REG_) CD4 T cells, and naïve and EM CD8 T cells. **(B)**. ICOS, CD44 and CTLA-4 mean fluorescence intensity (MFI) for *Ptprc^a/a^* versus *Ptprc^b/b^* T_REGS_ of the indicated genotypes (recipients received bone marrow from *n*=1 *Ptprc^b/b^ Card11^+/+^* donor and *n*=2 *Ptprc^b/b^ Card11^M365K/M365K^* donors). **(C)**. Representative flow cytometric histograms showing distribution of intracellular FoxP3 and CTLA-4 and cell-surface CD44 and ICOS fluorescence, for splenic *Ptprc^a/a^ Card11^+/+^* (black line) versus *Ptprc^b/b^ Card11^M365K/M365K^* (red line) T_REGS_. **(D)**. CD44^+^ CD62L^-^, CD44^+^ CD62L^+^ and CD44^-^ CD62L^+^ *Ptprc^a/a^* or *Ptprc^b/b^* T_REGS_, as a percentage of splenic CD4 T cells. Statistical comparisons made by paired *t*-test. not significant (n.s) p > 0.05; * *p* < 0.05; ** *p* < 0.01; *** *p* < 0.001.

To test whether *Card11^M365K^* acts cell-autonomously to dysregulate CD8 and CD4 T cells, we analyzed mixed chimeric mice containing *Card11^+/+^* CD45.1^+^ bone marrow-derived hematopoietic cells and CD45.2^+^ bone marrow-derived hematopoietic cells that were either *Card11^+/+^* or *Card11^M365K/M365K^*. Within individual chimeric mice, there was a significant increase in frequency of *Ptprc^b/b^ Card11^M365K/M365K^* relative to *Ptprc^a/a^ Card11^+/+^* CD4 effector memory (EM), T_FH_, T_REG_ and CD8 EM cells – whereas no such difference was observed between *Ptprc^b/b^ Card11^+/+^* and *Ptprc^a/a^ Card11^+/+^* cells (**Figure 5A**). *Card11^M365K/M365K^* thus provides a cell-intrinsic advantage to effector CD8 and CD4 T cells, T_FH_ and T_REG_ cells. The accumulating *Card11^M365K/M365K^* T_REGS_ had a significant cell-intrinsic increase in levels of cell-surface ICOS and CD44 and of intracellular CTLA-4 (**Figures 5B, C**). Within T_REG_ cells, and reminiscent of observations in germline *Card11*-mutant mice, *Card11^M365K/M365K^* mutation caused significant cell-autonomous accumulation of CD62L^-^ CD44^+^ T_REGS_ (**Figure 5D**).

Given the above findings, we tested whether *Card11^M365K^* mutation increases T cell responses to TCR, CD28 or high-affinity IL-2 receptor stimulation, which engage pathways crucial to the differentiation, survival and proliferation of effector T cells and T_REGS_. Following CTV labelling and 3 days of stimulation with anti-CD3 and anti-CD28 *in vitro*, the mean percentage of divided cells was 74% for *Card11^M365K/M365K^*, 68% for *Card11^M365K/+^* and 58% for *Card11^+/+^* CD4 T cells, and 94%, 90% and 87% for CD8 T cells of the respective genotypes. By contrast, only 12% and 24% for *Card11^loco/loco^* CD4 and CD8 T cells, respectively (**Supplementary Figure 4A**). *Card11^M365K^* mutation also increased and *Card11^loco/loco^* mutation decreased the size and cell-surface CD25 and PD-1 levels of stimulated CD4 and CD8 T cells (**Supplementary Figure 4B, C**). Given the small number of WT CD4 T cells assessed, we were unable to conclude that these effects were statistically significant. Our flow cytometric analysis of Ki67 expression revealed an increased fraction of Ki67+ T cells within the spleens of *Card11^M365K/M365K^* mutant mice (**Supplementary Figure 4D**). Thus, GOF CARD11.M365K caused increased activation and a mild increase in proliferation of CD4 and CD8 T cells following TCR stimulation and CD28 co-stimulation. By contrast, *Card11^M365K/M365K^* mutation had no effect on STAT5 phosphorylation following IL-2 stimulation (**Supplementary Figure 4E**). To determine the effects of CARD11 GOF on CD4 T cell differentiation *in vitro*, we purified naïve CD4 T cells from wild-type and mutant mice by fluorescence-activated cell sorting (FACS), and incubated them for 4 days in conditions that skew towards T helper 0 (T_H_0), T_H_1, T_H_2 or T_H_17 differentiation. At day 4, we observed increased frequencies of *Card11^M365K/M365K^* relative to *Card11^+/+^* IL-4^+^ and IL-5^+^ T_H_2-like cells (**Supplementary Figure 4F**). These results indicate that weak CARD11 GOF may skew naïve CD4 T cells towards T_H_2 differentiation in response to TCR and cytokine stimulation.

Collectively, these results demonstrate that GOF CARD11 increases T cell activation and proliferation following TCR and CD28 stimulation and provides a cell-intrinsic advantage to activated CD8 and CD4 T cells, T_FH_, T_REG_ and T_FR_ cells expressing increased levels of checkpoint molecules ICOS and PD-1.

Notably, PD-1 acts as a tumor suppressor in CD4 T cells (71), but PD-1 checkpoint therapy significantly worsens disease progression in some (72) but not all (73) individuals with ATL. ATL, which are thought to arise from effector and/or FoxP3^+^ CD4 T cells (74, 75), harbor recurrent somatic GOF *CARD11* mutations (38, 39). To test whether PD-1 restrains the over-accumulation of *Card11^M365K^* mutant CD4 T cells *in vivo*, we adapted a workflow used by Wartewig et al. to demonstrate that PD-1 inhibition synergises with *ITK-SYK* fusion to cause lethal CD4 T cell lymphoproliferation (71). We adoptively transferred 4 x 10^6^ *Ptprc^b/b^* CD4 T cells that were either *Card11^+/+^* or *Card11^M365K/M365K^*, into *Ptprc^a/a^ Card11^+/+^* C57BL/6 recipient mice. We injected the recipient mice with anti-PD-1 monoclonal antibody (mAb) or isotype control mAb at days 1, 3, 5 and 6, and sacrificed them at day 7 post-adoptive transfer (**Supplementary Figure 5A**). Consistent with our mixed chimera results, a higher fraction of *Card11*-mutant relative to wild-type CD4 T cells had an effector memory phenotype (**Supplementary Figure 5B**). Relative to control mAb-treated mice, anti-PD-1-treated mice had similar total numbers of cells per spleen (**Supplementary Figure 5C**). Amongst mice that received *Card11^M365K/M365K^* CD4 T cells, anti-PD-1 treatment increased the total number of donor-derived CD4 T cells, and resulted in a trend towards increased number of donor-derived effector memory CD4 T cells (**Supplementary Figure 5D, E**). These results indicate that PD-1 inhibition is insufficient to cause lymphoma or lethal lymphoproliferation of *Card11^M365K/M365K^* CD4 T cells, but that PD-1 may play a role in restraining the accumulation of *Card11^M365K/M365K^* CD4 T cells *in vivo*.

## 4 Discussion

The findings here reveal that gain-of-function mutation of a conserved CARD11 residue, located in the coiled-coil domain recurrently mutated in B- and T-lymphomas, caused cell-autonomous accumulation of effector CD8 and CD4 T cells, and particularly of T_FH_, T_REG_ and T_FR_ cells that are critical to coordinating and regulating adaptive immune responses. Germline *Card11^M365K^* GOF caused accumulation of spontaneous GC B cells and increased GC response to immune challenge but caused no dramatic B cell lymphocytosis as observed in individuals with BENTA disease. Our results demonstrate that CARD11 GOF perturbs T cells by increasing their activation and proliferation downstream of TCR or co-stimulatory receptor signaling. By revealing that GOF CARD11 drives aberrant expression of checkpoint molecules including PD-1 and ICOS, a known positive regulator of T_FH_, T_REG_ and T_FR_ cells, the findings here indicate that GOF *CARD11* mutations perturb T lymphocytes by dysregulating not only TCR-NFκB signaling but also co-stimulatory signaling. These results highlight likely effects of acquired GOF *CARD11* mutations that are strikingly recurrent in aggressive human PTCL derived from effector, follicular and regulatory CD4 T cells.

The mild increase in B cell numbers in *Card11^M365K^* mutant mice contrasts with lethal B cell lymphoproliferation upon B cell-conditional *Card11^L232LI^* mutation (*Card11^L225LI^* in the original publication (52);). This latter phenotype is also absent from mice with a *Card11^E134G^* or *Card11^K215M^* (54) or *Card11^L251P^* mutation (53). *Card11^K215M^* creates a cell-intrinsic advantage whereas *Card11^E134G^* creates a cell-intrinsic *disadvantage* for GC B cells (54), and *Card11^L251P^* acts primarily to alter GC kinetics (53). The variable effects on B cells of different *CARD11* mutations may relate to qualitative differences in their effect on NF-κB signaling activity (31, 55). In a luciferase reporter system in 293T cells, CARD11 p.M365K increased the transcription of a NF-κB target gene to levels above wild-type CARD11, but below CARD11 p.L251P (30). Our results, based on a NF-κB luciferase assay, B220 and CD86 expression and survival of B cells transduced with *CARD11^WT^*, *CARD11^G123S^*, *CARD11^E134G^* and *CARD11^M365K^*, indicate that *CARD11^M365K^* leads to weak GOF intermediate between that caused by *CARD11^E134G^* and *CARD11^G123S^*.

The *Card11^M365K^* mutant mouse strain was generated by ENU mutagenesis, which enabled us to study the effects of *Card11* GOF mutation in an otherwise normal gene, as opposed to expression of mutant *Card11* cDNA from a heterologous promoter and locus (52, 53, 76). In this context, *CARD11^M365K^* was insufficient to cause B cell malignancy or the striking B cell lymphocytosis seen in individuals with BENTA disease. This contrast may relate to differing *CARD11* mutations, as discussed above and given that *CARD11^M365K^* has not been identified in the germline of children with BENTA disease. Alternatively, the contrast may relate to the specific-pathogen free environment of the *Card11^M365K^* mice or to differences between human and mouse lymphocytes. Like germline GOF *CARD11* mutations, *BTK, NFKB1* or *NFKB2* deficiency have different consequences in humans relative to mice. In humans, they cause profound loss of transitional and mature naïve B cells (77–80) but in mice they cause a less drastic decrease (81–84), indicating that human B cells may be more strongly dependent on BTK-NF-κB signaling. Several hypotheses may explain the profound increase in transitional and mature B cells caused by germline GOF *CARD11* mutations in humans but not mice. (i) *Card11* mRNA increases 10-fold between pre-B cells and immature IgM^+^ B cells and transitional B cells in mice (Immgen Database). It is possible that CARD11 mRNA and protein are more strongly expressed in human relative to mouse naïve B cells, beyond a threshold where GOF in the protein dysregulates proliferation and survival. Nevertheless, previous publications (52, 54, 76) and our *in vitro* data indicate that GOF CARD11 can provide a cell-intrinsic advantage to mouse B cells. (ii) CARD11 mRNA or protein may be down-regulated in mouse B cells as an adaptive response to GOF CARD11 signaling that does not function in human B cells. (iii) Human B cells may be less able to induce counter-regulatory processes acting downstream or upstream from CARD11 (i.e. induction of NFKBIA or TNAIP3). Future studies comparing CARD11, NFKBIA and TNFAIP3 protein levels in normal and *CARD11*-mutant human and mouse transitional and mature B cells may help to distinguish between these alternatives.

T cells from human BENTA patients carrying CARD11 GOF mutations typically proliferate less than healthy controls in response to anti-CD3/CD28 stimulation, a difference linked to a mildly anergic phenotype associated with poor IL-2 expression by *CARD11*-mutant human T cells (29). By contrast, *Card11^M365K^* mutant mouse T cells had a mild proliferative advantage relative to Card11 wild-type mouse T cells. In addition to the considerations discussed above, it is possible that secondary effects that are visible in humans over time may not be visible in mice at 8-12 weeks of age. (i) These secondary effects may be pathological, as seen in *CTLA4* deficiency in humans, which results in loss of B cells even though B cells mostly lack CTLA4 expression. Affected patients have relatively normal B cell numbers prior to developing pathology but start losing B cells when they develop the syndrome (85). (ii) These secondary effects may be compensatory, as seen in transgenic B cells expressing chimeric IgMG receptors containing the IgG tail segment. These cells adopt a gene expression profile of anergy, but this occurs secondary to their down-regulation of cell-surface receptor (86).

The skewing of towards Th2 differentiation of *Card11^M365K/M365K^* CD4 T cells is interesting, given that Th2 skewing occurs in humans with loss-of-function or dominant negative *CARD11* mutations. We cannot exclude the possibility that CARD11.M365K results in “blended” GOF and LOF effects, as previously observed in BENTA disease (26). Mice homozygous for the *hypomorphic Card11^unmodulated^* mutation develop penetrant, spontaneous atopy and dermatitis with age (10), caused by partial reduction in effector T cell accumulation but also partial T_REG_ deficiency leading to progressive, selective T_H_2 accumulation and subsequent IgE production (16). In that context, hypomorphic *Card11* mutation produces outcomes that could not be predicted from null alleles, through unequal titration of opposing effects within different T cell subsets (16). Similarly, unequal effects of *hypermorphic* mutations in different lymphocyte populations may contribute to the variable B and T cell pathologies in humans and mice with germline and somatic *CARD11* GOF mutations.

In addition to cell-intrinsic effects, B cell homeostasis may be perturbed by CARD11 GOF within CD4 T cells. Previous publications studied *Card11^L251P^* (53) and *Card11^L232LI^* (52) expressed in B cells only, whilst T cell populations from germline *Card11^E134G^* and *Card11^K215M^* mutant mice were not reported (54). In *Card11^M365K^* mutant mice, the accumulation of splenic T_FH_ cells at days 7, 12 and 15, but not at day 5 post-immunization, correlated with accumulation of splenic GC B cells at days 7, 12 and 15, but not at day 5. *Card11^M365K^* mutant T_FH_ cells expressed homogeneously increased levels of cell-surface ICOS, and both T_FH_ accumulation and increased ICOS expression (87) are known to drive GC B cell accumulation. *Card11^M365K/M365K^* mutation caused cell-autonomous accumulation of ICOS^high^ T_FH_ cells, but also of T_FR_ cells that can act to suppress the GC response (88–90). The relative, and possibly graded, effects of *Card11* mutation in T_FH_ versus T_FR_ cells, and in turn on B cell homeostasis, are difficult to distinguish without T_FH_ or T_FR_-specific CARD11 GOF models. Nevertheless, our data raise the possibility that CARD11 GOF CD4 T cells may perturb B cells in individuals with germline or somatic *CARD11* GOF mutations. Individuals with BENTA disease have normal numbers of circulating CD4 and CD8 T cells (25, 29), but to our knowledge no detailed T cell immunophenotyping has been reported. Future studies should assess T cell populations in humans and mouse models with different germline or T cell-restricted *CARD11* mutations.

With regards to T cell lymphoma, our results reveal likely cell-intrinsic effects of the somatic GOF *CARD11* mutations that recur in up to 30% of ATL (38, 39), CTCL and Sezary Syndrome (43–46) and at lesser frequency in angioimmunoblastic T cell lymphoma (AITL) (91). *CARD11* and *PRKCB* mutations are positively correlated in ATL (38), suggesting that NF-κB activating mutations may synergize in driving ATL. The striking recurrence of mutations modifying the TCR/NF-κB pathway highlights its importance in PTCLs including ATL (38, 39, 56–58) and CTCL or Sezary syndrome (43–46, 59–61, 92, 93). One limitation of our study is that *CARD11^M365K^* has not been identified in PTCL or CTCL. Nevertheless, *CARD11^M365K^* modifies a conserved region of the CC domain recurrently mutated in PTCL (**Figure 1**). ATL, CTCL and AITL are thought to arise from activated, T_FH_-like and/or T_REG_-like CD4 T cells (50, 94–96), and *Card11^M365K^* mutation causes cell-autonomous accumulation of activated, T_FH_, T_REG_ and T_FR_ CD4 T cells. In addition, *Card11^M365K/M365K^* caused over-expression of stimulatory and inhibitory receptors ICOS, CTLA-4 and PD-1, and increased activation, proliferation and PD-1 expression by mutant T cells following TCR and CD28 stimulation. Activating *CD28* mutations recur in 10-11% of AITL (97, 98), and in-frame fusions involving *CD28, CTLA4* and *ICOS* recur in 7% of ATL along with *CD28* focal gains and missense mutations, all of which result in continuous or prolonged co-stimulatory signaling (38). When expressed in mice on a *Tet2^-/-^* background, the *RHOA^G17V^* mutation identified in 70% of AITL (99–101) results in T cell lymphomas that partially require ICOS and PI3K signaling for their proliferation and survival (102). The cell-intrinsic increase of ICOS and CTLA-4 expression on *Card11^M365K^* mutant CD4 and T_REG_ cells indicates that CARD11 GOF may contribute to CD4 T cell dysregulation not just *via* TCR-NFκB but also *via* PI3K signaling. ICOS expression increases the accumulation of T_FH_ cells but also of T_REG_ and T_FR_ cells (103), such that ICOS over-expression on expanded *CARD11^M365K^* mutant T_FH_, T_REG_ and T_FR_ cells may further their accumulation. By contrast, increased CTLA-4 on the surface of these *Card11*-mutant cells may limit their accumulation (104).

In addition to ICOS and CTLA-4, *Card11^M365K^* mutation increased PD-1 expression by CD4 T cells, *in vivo* and following TCR/CD28 stimulation *ex vivo*. Parallel observations could be drawn by future studies testing the association of *CARD11* mutations with increased PD-1 or ICOS expression on human T-lymphoma cells. *PDCD1* (encoding PD-1) is increased in CD4 malignancies with gene signatures of dysregulated TCR signaling (71). PD-1 acts as a tumor suppressor in CD4 T cells and *PDCD1* alterations, most commonly focal deletions, recur in 10-20% of CTCL, 36% of Sezary syndrome and 26% of ATL (71). Consistent with the effects of PD-1 in inhibiting TCR signaling and also CD28 co-stimulation (105), PD-1 inhibition mildly increased *Card11^M365K/M365K^* CD4 T cell accumulation *in vivo*, but was nevertheless insufficient to cause CD4 lymphoma or lymphoproliferation. This contrasts with the lethal lymphoproliferation of CD4 T cells expressing an *ITK-SYK* fusion upon their exposure to anti-PD-1 monoclonal antibody (71). This dichotomy may point to a threshold of CARD11 or NF-κB GOF required for synergy with PD-1 LOF to drive CD4 lymphoproliferation. The acquisition of different somatic driver gene mutations (e.g. *ITK-SYK* fusion versus intermediate *CARD11* GOF mutation) may explain why PD-1 inhibition accelerates disease progression in some (72) but not all (73) cases of ATL.

Unlike other PTCL, ATL requires HTLV-1 infection (41, 106, 107). The variable, often long, latent phase between HTLV-1 infection and ATL diagnosis implicated additional environmental or genetic events in ATL pathogenesis, and led to the discovery of TCR/NF-κB pathway genes and *CARD11* as recurrently mutated in ATL (38, 39). Notably, HTLV-1 viral proteins TAX and HBZ increase NF-κB activation and survival (108, 109), and HTLV-1 has tropism for FoxP3^+^ CD4 T cells (109–111). Given that *Card11^M365K^* mutation increases NF-κB activation and creates a cell-intrinsic advantage for T_REGS_, the cell-intrinsic effects of *CARD11* mutations and of HTLV-1 infection may cooperate in driving ATL pathogenesis.

There is a striking paucity of information on the cell-intrinsic effects of somatic *CARD11* GOF mutations in PTCL, which are heterogeneous and often aggressive malignancies associated with poor clinical outcomes (112). The above findings reveal cell-intrinsic effects of a CARD11 GOF protein within T cells. They highlight the need to study T cells in humans with germline *CARD11* GOF mutations and BENTA disease, and in mouse models with PTCL hotspot *CARD11* mutations. Our findings further highlight the crucial role played by CARD11 in lymphocytes and the possible therapeutic utility of developing small molecule inhibitors targeting CARD11.

## Data availability statement

The original contributions presented in the study are included in the article/**Supplementary Material**. Further inquiries can be directed to the corresponding authors.

## Ethics statement

The animal study was reviewed and approved by Garvan Institute of Medical Research/St Vincent’s Hospital Animal Ethics Committee; ANU National University Animal Experimentation Ethics Committee.

## Author contributions

EM-F and YJ designed and performed the experiments. JW, RB and KH designed and/or performed transduction experiments. JB and ED performed the Th differentiation assay. EM-F, YJ, KH and CG interpreted experiments and wrote the manuscript. All authors contributed to the article and approved the submitted version.
