# Supplementary material for: CARD11 gain-of-function mutation drives cell-autonomous accumulation of PD-1+ ICOShigh activated T cells, T-follicular, T-regulatory and T-follicular regulatory cells
Source: Front Immunol. 2023 Mar 7;14:1095257. doi: 10.3389/fimmu.2023.1095257 (PMC10028194; doi:10.3389/fimmu.2023.1095257)
Supplement: Supplementary file 1 [file DataSheet_1.pdf]

Supplementary Material

1 Supplementary Figures

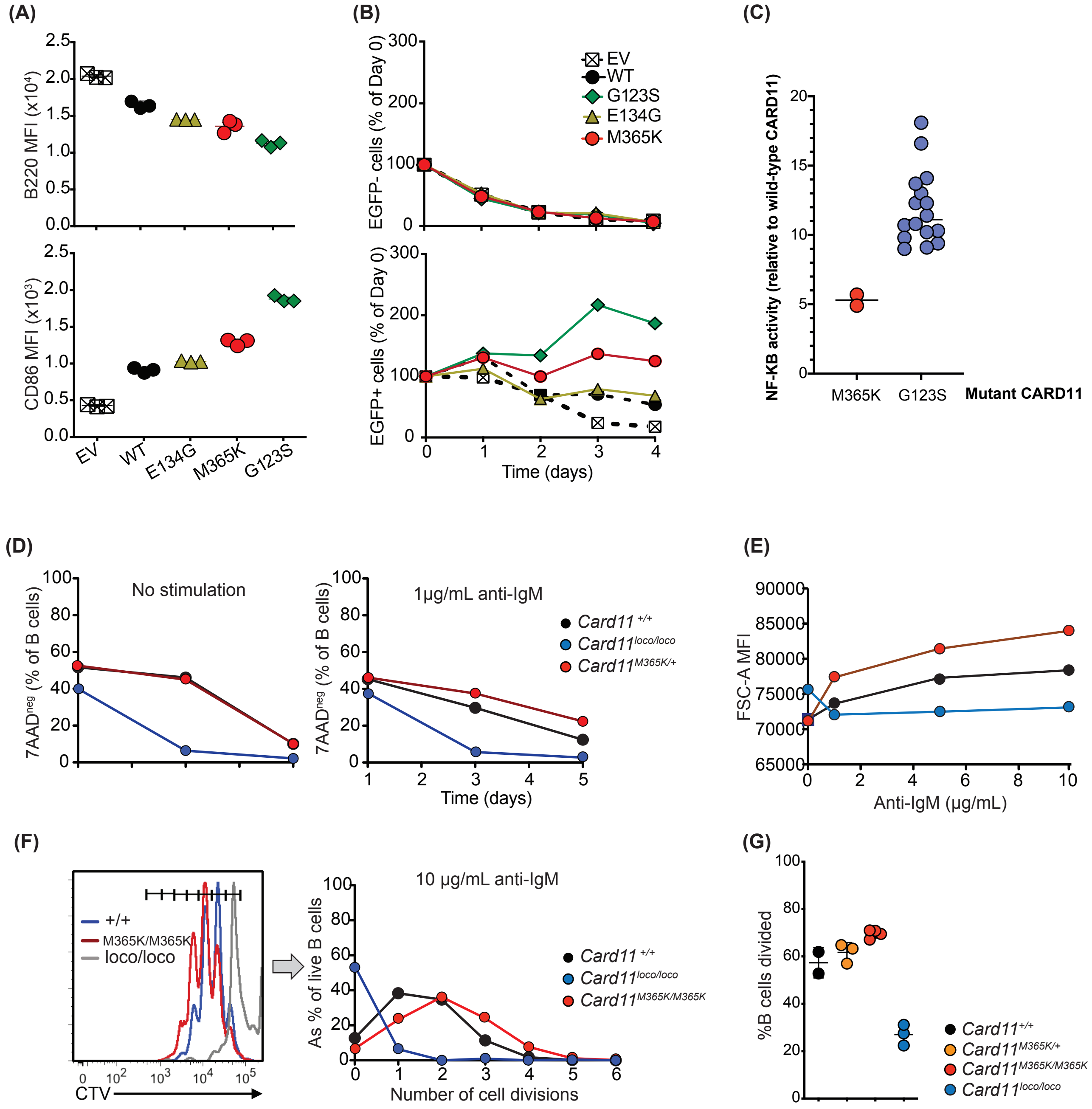

Figure S1. CARD11.M365K is a weak to intermediate gain-of-function protein that increases B cell proliferation following BCR stimulation.

(A,B). C57BL/6 mouse primary B cells were stimulated with 10 μg/mL goat anti-mouse IgM F(ab')$_2$ and 10 μg/mL anti-CD40, and transduced with retroviruses containing pMX-IRES-GFP empty vector, wild-type *Card11* (circles; black fill), *Card11*^{E134G} (triangles; brown fill), *Card11*^{G123S} (diamonds; green fill) or *Card11*$^{M365K/M365K}$ (circles; red fill). (**A**)**.** Mean fluorescence intensity (MFI) for B220 (top) and CD86 (bottom) on activated B cells transduced with the indicated vectors. Data are representative technical triplicates for each condition. (**B**)**.** Live (7AAD-) EGFP^{neg} (top) or EGFP^{pos} (bottom) cells expressed as a percentage over time of the B cell number at day 0, for B cells transduced with the indicated vectors. (**C**)**.** NF-κB activation by CARD11 mutants relative to activation by wild-type CARD11, as measured by luciferase reporter assay. Each point represents an independent experiment, and bars show mean + SD. (**D-F**)**.** Primary B cells from *Card11*$^{+/+}$ (black), *Card11*$^{loco/loco}$ (blue) or *Card11*$^{M365K/+}$ (orange) mice were cultured in complete RPMI (cRPMI) containing various concentrations of anti-mouse IgM F(ab')$_2$. (**D**)**.** Survival (based on 7AAD staining) of mouse B cells of the indicated genotypes, following culture in RPMI only (top) or in the presence of a sub-mitogenic 1μg/mL dose of anti-IgM F(ab'$_2$) (bottom). (**E**)**.** Size, based on blue laser forward scatter (FSC-A), of mouse B cells cultured for 3 days in the presence of the indicated concentrations of anti-IgM F(ab'$_2$). (**F**)**.** Representative histogram overlay of CTV fluorescence (left), and enumeration of cell divisions (right), for mouse B cells stimulated for 3 days in the presence of 10 μg/mL anti-IgM F(ab'$_2$). **(G).** Percentage of B cells having undergone at least one cell division, following 3 days of *in vitro* anti-IgM (10ug/mL) stimulation of lymphocytes from *Card11*$^{+/+}$ (black fill), *Card11*$^{M365K/+}$ (orange fill), *Card11*$^{M365K/M365K}$ (red fill) or *Card11*$^{loco/loco}$ (blue fill) mice. Each symbol is the average of n = 3 technical triplicates. **(A)** Comparisons made by t-test, corrected for multiple comparisons using the Holm-Sidak method. * $p < 0.05$; ** $p < 0.01$; *** $p < 0.001$.

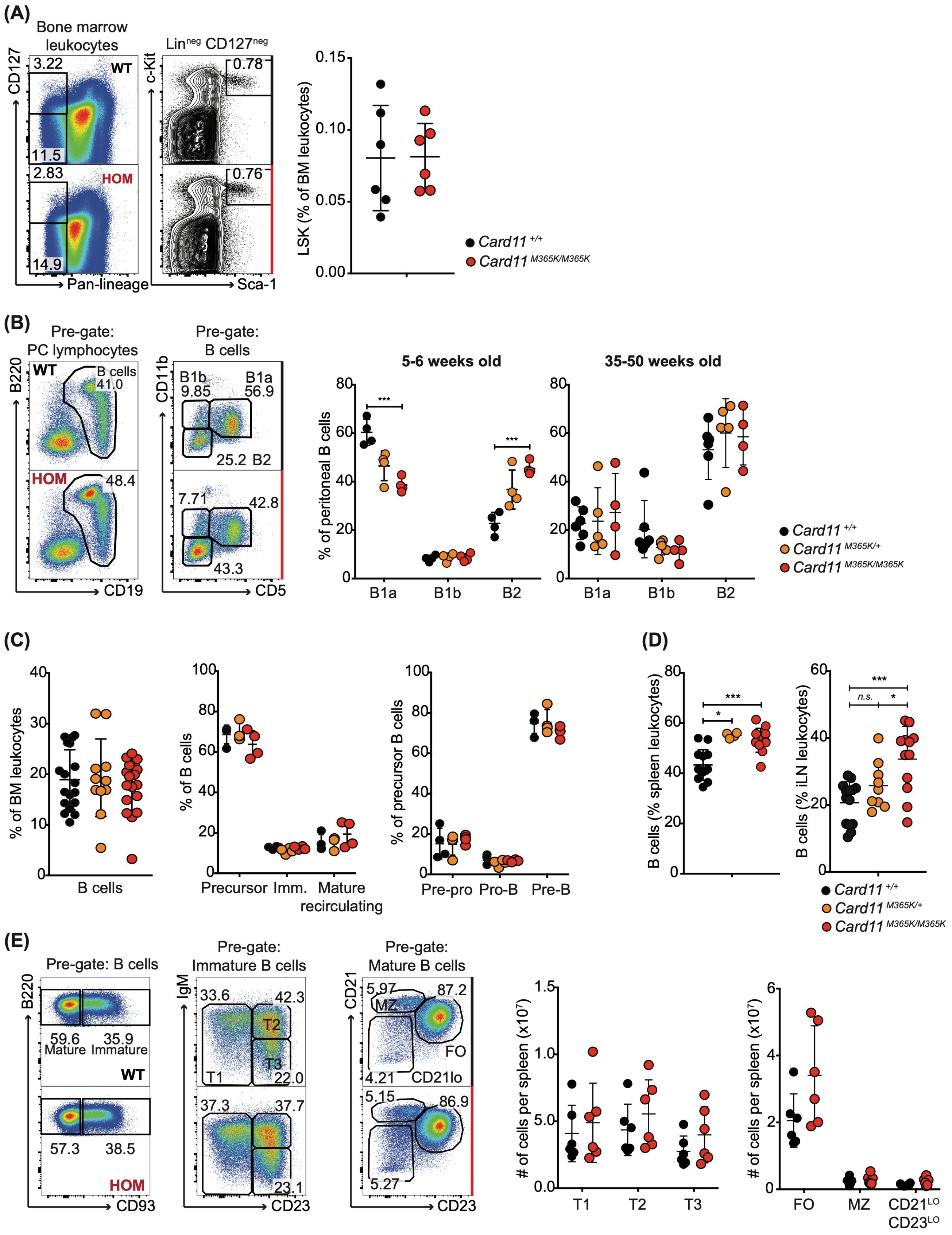
(A)
Bone marrow leukocytes
CD127
3.22
WT
11.5
2.83
HOM
14.9
Pan-lineage
Lin^neg CD127^neg
c-Kit
0.78
0.76
Sca-1
LSK (% of BM leukocytes)
0.15
0.10
0.05
0.00
Card11 +/+
Card11 M365K/M365K
(B)
Pre-gate: PC lymphocytes
B220
WT
B cells
41.0
HOM
48.4
CD19
Pre-gate: B cells
CD11b
B1b
9.85
B1a
56.9
25.2 B2
7.71
42.8
43.3
CD5
5-6 weeks old
% of peritoneal B cells
***
***
B1a
B1b
B2
35-50 weeks old
Card11 +/+
Card11 M365K/+
Card11 M365K/M365K
(C)
% of BM leukocytes
B cells
% of B cells
Precursor
Imm.
Mature recirculating
% of precursor B cells
Pre-pro
Pro-B
Pre-B
(D)
B cells (% spleen leukocytes)
***
*
B cells (% iLN leukocytes)
***
n.s.
*
Card11 +/+
Card11 M365K/+
Card11 M365K/M365K
(E)
Pre-gate: B cells
B220
59.6
Mature
35.9
Immature
WT
57.3
38.5
HOM
CD93
Pre-gate: Immature B cells
IgM
33.6
42.3
T2
T3
T1
22.0
37.3
37.7
23.1
CD23
Pre-gate: Mature B cells
CD21
5.97
MZ
87.2
FO
4.21
CD21lo
5.15
86.9
5.27
CD23
# of cells per spleen (x10^7)
T1
T2
T3
# of cells per spleen (x10^7)
FO
MZ
CD21^LO CD23^LO

Figure S2. Hematopoietic stem cells and B cell populations in the peritoneal cavity, bone marrow and spleen of *Card11^{M365K}* mice.

(A). Left, representative flow cytometric analysis of lineage negative (Lin$^-$) CD127$^-$ c-Kit$^+$ Sca-1$^+$ (LSK) bone marrow hematopoietic stem cells. Right, LSK cells as a percentage of bone marrow leukocytes, in mice of the indicated genotypes. Data are representative of $n = 2$ independent experiments with $n > 5$ mice per group. (**B).** Left, representative flow cytometric analysis of peritoneal cavity (PC) CD19$^+$ B cell subsets: CD5$^+$ CD11b$^+$ B1a, CD5$^+$ CD11b$^-$ B1b and CD5$^-$ CD11b$^-$ B2. Right, B1a, B1b and B2 cells as a percentage of PC B cells in mice of the indicated genotypes and ages. (**C).** Percentage of bone marrow (BM) B cells, IgM$^-$ IgD$^-$ precursor, IgM$^+$ IgD$^-$ immature and IgM^{int} IgD$^+$ mature recirculating B cells, and CD43^{high} CD24$^-$ pre-pro-, CD43^{int} CD24^{int} pro- and CD43^{low} CD24$^+$ pre-B cells, in mice of the indicated genotypes. (**D).** B cells as a percentage of spleen (left) or inguinal lymph node (iLN; right) leukocytes, in mice of the indicated genotypes. (**E).** Left, representative flow cytometric analysis of splenic CD93$^+$ immature CD23$^-$ T1, CD23$^+$ IgM$^+$ T2, CD23$^+$ IgM$^-$ T3 and CD93$^-$ mature CD23$^+$ follicular (FO), CD23^{low} CD21$^+$ marginal zone (MZ) and CD23^{low} CD21^{low} B cells. Right, total number per spleen of T1, T2, T3, FO, MZ and CD21^{low} CD23^{low} B cells, in *Card11$^{+/+}$* (black) or *Card11$^{M365K/M365K}$* (red) mice. **(A-E)** Statistical comparisons made by *t*-test, corrected for multiple comparisons using the Holm-Sidak method. * $p < 0.05$; ** $p < 0.01$; *** $p < 0.001$.

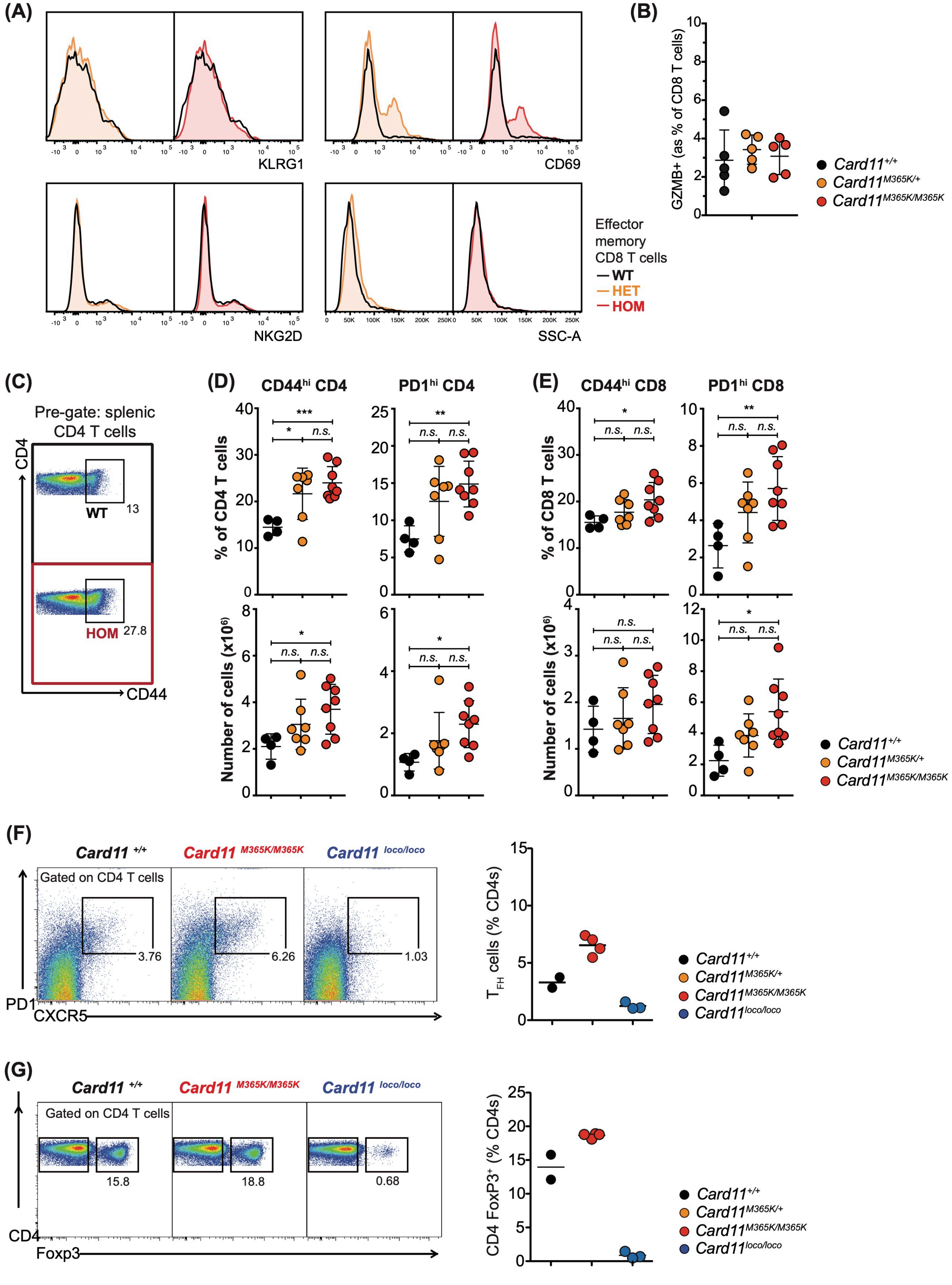

(A)
KLRG1
CD69
NKG2D
SSC-A
Effector memory CD8 T cells
WT
HET
HOM
(B)
GZMB+ (as % of CD8 T cells)
Card11+/+
Card11M365K/+
Card11M365K/M365K
(C)
Pre-gate: splenic CD4 T cells
CD4
CD44
WT 13
HOM 27.8
(D)
CD44hi CD4
PD1hi CD4
% of CD4 T cells
Number of cells (x10^6)
(E)
CD44hi CD8
PD1hi CD8
% of CD8 T cells
(F)
Card11 +/+
Card11 M365K/M365K
Card11 loco/loco
Gated on CD4 T cells
3.76
6.26
1.03
PD1
CXCR5
TFH cells (% CD4s)
Card11loco/loco
(G)
15.8
18.8
0.68
Foxp3
CD4 FoxP3+ (% CD4s)

Figure S3. Increased activated CD4 and CD8 T cells, T follicular helper-like and T regulatory cells in *Card11^{M365K}* mutant mice on a C57BL/6 Ncrl background.

(**A**)**.** Representative histogram overlays of cell-surface KLRG1, CD69, NKG2D expression and side scatter as a measure of granularity, for splenic effector memory CD8 T cells of the indicated genotypes. (**B**)**.** Percentage of granzyme-B positive splenic CD8 T cells in mice of the indicated genotypes. **(C-G).** A small number of experiments were performed on *Card11^{M365K}*-mutant mice on a C57BL/6 Ncrl background, as opposed to all other experiments in this manuscript performed on mice from a C57BL/6 JAusb background. (**C**)**.** Representative flow cytometric analysis of CD44 expression on splenic CD4 T cells of a *Card11$^{+/+}$* (top) or *Card11$^{M365K/M365K}$* (bottom) mouse. (**D**)**.** CD44^{high} (left) or PD-1^{high} (right) cells as a percentage of splenic CD4 T cells or as a total number per spleen, in mice of the indicated genotypes. (**E**)**.** CD44^{high} (left) or PD-1^{high} (right) cells as a percentage of splenic CD8 T cells or as a total number per spleen, in mice of the indicated genotypes. (**F**)**.** Representative flow cytometric analysis of CXCR5 and PD-1 expression, and percentage of CXCR5^{high} PD-1^{high} TFH-like CD4 T cells, in mice of the indicated genotypes. (**G**)**.** Representative flow cytometric analysis of FoxP3 and CD4 expression, and percentage of CD4^{+} FoxP3^{+} T regulatory cells, in mice of the indicated genotypes. **(B,D,E).** Statistical comparisons made by *t*-test, corrected for multiple comparisons using the Holm-Sidak method. * $p < 0.05$; ** $p < 0.01$; *** $p < 0.001$.

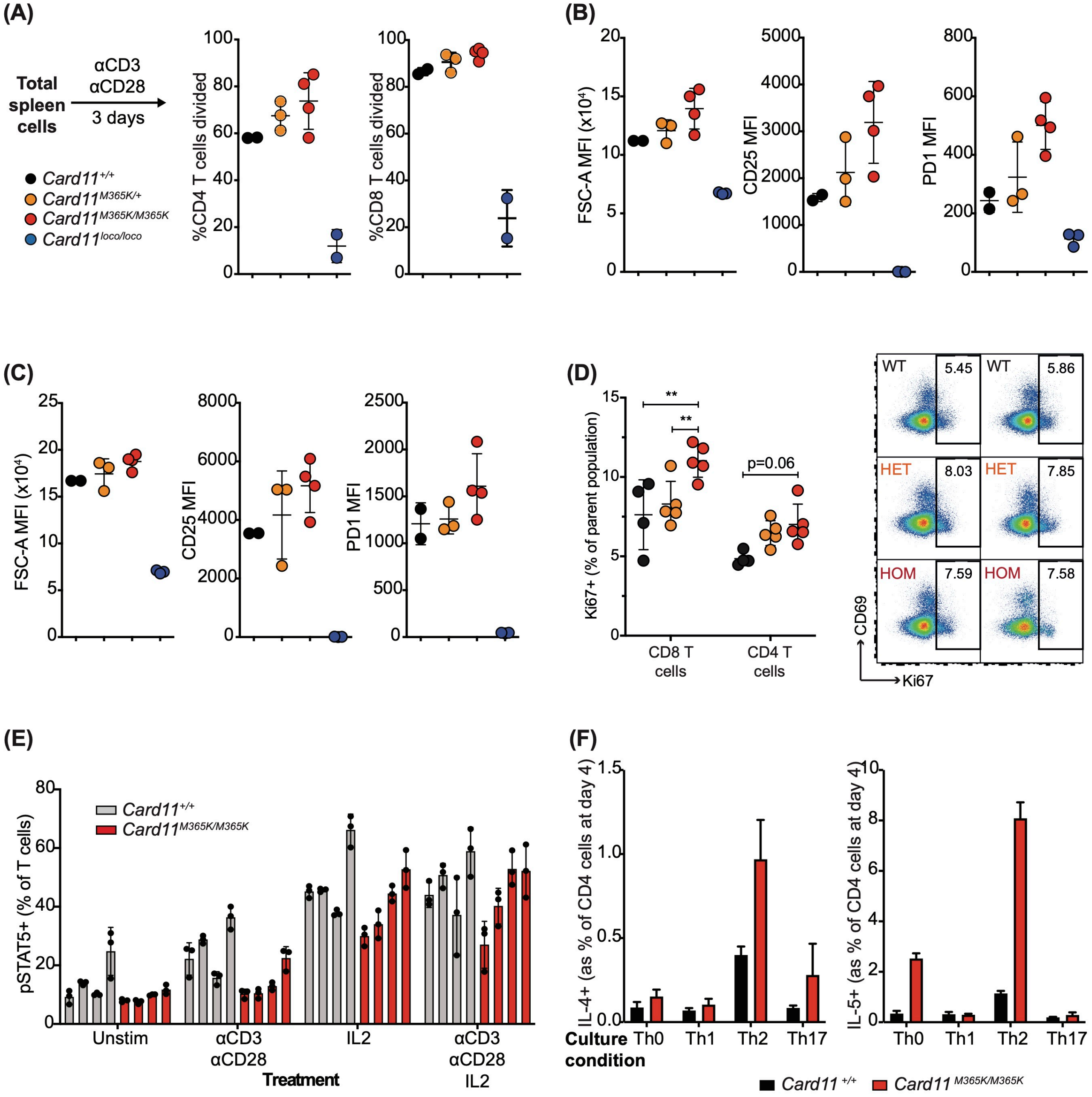
(A)
Total spleen cells
αCD3 αCD28
3 days
Card11+/+
Card11M365K/+
Card11M365K/M365K
Card11loco/loco
%CD4 T cells divided
%CD8 T cells divided
(B)
FSC-A MFI (x10⁴)
CD25 MFI
PD1 MFI
(C)
FSC-A MFI (x10⁴)
CD25 MFI
PD1 MFI
(D)
Ki67+ (% of parent population)
p=0.06
CD8 T cells
CD4 T cells
WT 5.45
WT 5.86
HET 8.03
HET 7.85
HOM 7.59
HOM 7.58
CD69
Ki67
(E)
pSTAT5+ (% of T cells)
Card11+/+
Card11M365K/M365K
Unstim
αCD3 αCD28
IL2
αCD3 αCD28 IL2
Treatment
(F)
IL-4+ (as % of CD4 cells at day 4)
IL-5+ (as % of CD4 cells at day 4)
Culture condition
Th0
Th1
Th2
Th17
Card11 +/+
Card11 M365K/M365K

Figure S4. Increased activation and proliferation of *Card11^{M365K}* mutant T cells following TCR stimulation and CD28 co-stimulation.

(A-C). Each symbol is the average of *n* = 3 technical triplicates, following 3 days of *in vitro* anti-CD3 and anti-CD28 stimulation of lymphocytes from *Card11$^{+/+}$* (black fill), *Card11$^{M365K/+}$* (orange fill), *Card11$^{M365K/M365K}$* (red fill) or *Card11$^{loco/loco}$* (blue fill) mice. (**A**)**.** Percentage of CD4 (left) or CD8 (right) T cells having undergone at least one cell division. (**B,C**)**.** Size, measured as blue laser forward scatter (FSC-A), cell surface CD25 or PD-1 expression by CD4 T cells (**B**) or CD8 T cells (**C**) from mice of the indicated genotypes. (**D**). Left, percentage of Ki67+ cells within splenic CD8 or CD4 T cells from *Card11^{M365K}* mice of the indicated genotypes; Right, representative flow cytometric analysis of Ki67 and CD69 expression by CD4 T cells from *Card11$^{+/+}$* (WT), *Card11$^{M365K/+}$* (HET) or *Card11$^{M365K/M365K}$* (HOM) mice. (**E**)**.** Percentage of pSTAT5-Y694^{+} T cells following 25 minutes incubation of splenic T cells from *Card11$^{+/+}$* (grey bars; *n* = 4) or *Card11$^{M365K/M365K}$* (red bars; *n* = 4) mice, with cRPMI alone or containing anti-CD3 and anti-CD28, or IL-2 or anti-CD3, anti-CD28 and IL-2. Each bar corresponds to cells from a separate animal; each circle denotes one of 3 technical triplicates. (**F**)**.** Percentage of CD4 T cells positive for intracellular IL-4 (left) or IL-5 (right), following 4 days incubation of sorted naïve CD4 T cells with Th0-, Th1-, Th2- or Th17-skewing conditions. Data were obtained from 3 mice per group, with 2 technical replicates per mouse per condition.

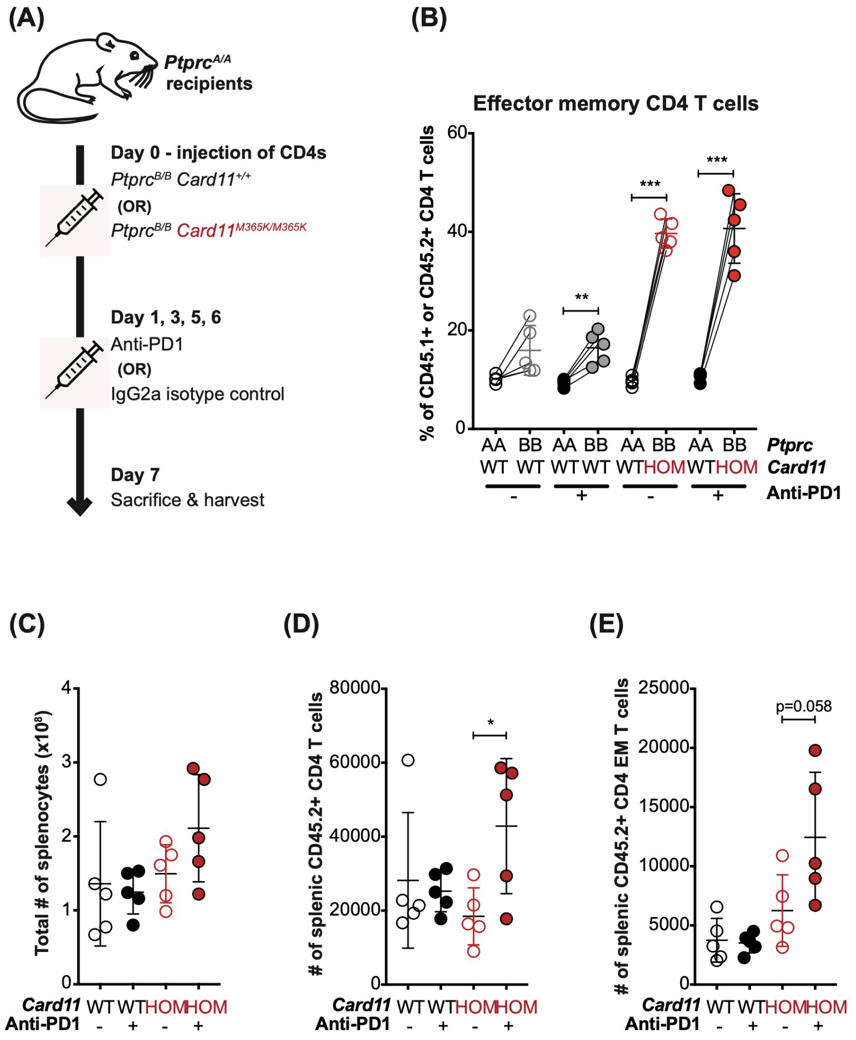

Figure S5. PD-1 inhibition using a neutralizing monoclonal antibody is not sufficient to cause lymphoproliferation of *Card11*$^{M365K/M365K}$ mutant CD4 T cells.

On day 0, C57BL/6 *Ptprc*$^{a/a}$ mice were injected with 4 x 10^6 CD4 T cells from *Card11*$^{+/+}$ or *Card11*$^{M365K/M365K}$ *Ptprc*$^{b/b}$ donor mice (n = 5 recipients per group), followed by *i.p.* injections on days 1, 3, 5, 6 of PBS containing 200 µg of anti-PD1 (clone RMP1-14) or isotype control IgG2a antibody. Mice were sacrificed 7 days post-adoptive transfer. (**A**). Schematic experimental workflow of the adoptive transfer experiment. (**B**). Pairwise comparison of the percentage of endogenous *Ptprc*$^{a/a}$ versus donor-derived *Ptprc*$^{b/b}$ CD4 T cells with a CD44^{+} CD62L^{-} effector memory phenotype. Black lines link cells from within the same recipient mouse. (**C**) Total number of leukocytes per spleen, (**D**) total number per spleen of *Ptprc*$^{b/b}$ donor-derived CD4 T cells and (**E**) total number per spleen of effector memory CD4 T cells, in mice that received the indicated cells and treatments. **(B)** Populations within the same chimeric mouse were compared by paired t-test. **(C-E).** Groups compared by t-test, corrected for multiple comparisons using the Holm-Sidak method. * $p < 0.05$; ** $p < 0.01$; *** $p < 0.001$.
